# A Focus on Thermal Durability and Oxidation Resistance and Morphology of Polymer Capped Copper Particles Through a Synthesis-Driven, Precursor-Influenced Approach

**DOI:** 10.3390/nano15241852

**Published:** 2025-12-10

**Authors:** A. R. Indhu, Manickam Minakshi, R. Sivasubramanian, Gnanaprakash Dharmalingam

**Affiliations:** 1 Plasmonic Nanomaterials Laboratory, Department of Nanoscience and Technology, PSG Institute of Advanced Studies, Coimbatore 641 004, Tamil Nadu, India; 2 College of Science, Health, Engineering and Education, Murdoch University, Perth, WA 6150, Australia; 3 Department of Chemistry, Amrita School of Physical Sciences, Amrita Vishwa Vidyapeetham, Amaravati 522 240, Andhra Pradesh, India; s_subramanian@av.amrita.edu; 4 Department of Physics and Nanotechnology, SRM Institute of Science and Technology, Kattankulathur, Chennai 603 203, Tamil Nadu, India

**Keywords:** oxidation-stable copper particles, polymer-capped copper particles and precursor-mediated copper oxidation stability

## Abstract

Copper is a promising alternative to conventional plasmonic materials, though its practical use is hindered by a strong tendency to oxidize. Through systematic analysis of its vibrational, optical, morphological, structural, and surface potential properties, we confirmed the stability of copper (Cu) particles and highlighted the role of functional groups in modulating their oxidation susceptibility. Oxidation kinetics at 150 °C, in the presence of antioxidants and capping agents, as well as long-term colloidal stability, appear closely tied to the degradation of these stabilizers, which correlates with particle aggregation. Notably, precursor chemistry significantly affects oxidation behavior. Varying concentrations of polyvinylpyrrolidone (PVP) demonstrate a positive correlation with particle size control and thermal stability, indicating that PVP enhances oxidation resistance under the tested conditions. Our findings underscore most importantly the metallic phase’s stability after exposure to air at a temperature of 150 °C, drawing attention to a possible precursor and capping agent combination that can result in oxidation-stable Cu particles, positioning them as cost-effective candidates for replacing more expensive plasmonic metals across diverse applications.

## 1. Introduction

Metal nanoparticles are central to materials research due to their tunable optical, chemical, and electrical properties, which can be precisely controlled by adjusting particle size [1], shape [2], and the surrounding medium [3]. Noble metals such as gold (Au), silver (Ag), palladium (Pd), and platinum (Pt) have received extensive attention over recent decades, primarily because of their stability and ease of modification compared to emerging alternatives like copper (Cu) [4].

To advance the use of Cu in plasmonic applications due to its interesting high dielectric loss, broadband absorbance and free electron density attributes compared to prominent plasmonic materials like Au, it is essential to address the challenge of synthesizing Cu particles that resist oxidation and agglomeration—issues that persist even under ambient storage conditions [5,6]. Upon oxidation, Cu primarily forms cuprous oxide (Cu_2_O) [7], though cupric oxide (CuO) and intermediate phases such as Cu_x_O (where x ≈ 0.67) [8] are also observed. Oxidation rates can follow a linear trend, with oxide film growth reported at 0.004 nm/day under ambient conditions [9] and up to 0.034 nm/day for 100–140 nm spherical CuNPs [10]. Some studies note a saturation effect, where a 13 nm oxide layer forms within hours, independent of oxygen pressure. Initially, uniform surface passivation was proposed as the dominant oxidation mechanism [11], but this was later revised to an island nucleation and coalescence model [12]. Although oxidation may appear self-limiting in air, this behavior vanishes at elevated temperatures (e.g., 50 °C), leading to complete oxidation [13]. The resulting loss of metallic Cu characteristics significantly alters its plasmonic properties [14,15,16]. Despite the variability in oxidation behavior due to multiple influencing factors [17], the issue remains critical. Various mitigation strategies have been explored, including alloying with other metals [18], forming composites with metal oxides [19] or polymers [20], and employing ligand functionalization [21]. Studies have demonstrated that ascorbic acid (AA) can enhance the oxidation stability of Cu nanoparticles (NPs), primarily through its reversible transformation to dehydroascorbic acid (DHAA) and back to AA via hydrolysis [22,23,24,25,26,27]. Our own findings support this role, showing that AA significantly improves the long-term oxidation resistance of CuNPs deposited on glass substrates, with particle size strongly influenced by AA concentration [28]. With its four hydroxyl groups, AA forms complex structures through intra- and intermolecular hydrogen bonding, acting as an effective antioxidant. However, AA alone is insufficient to maintain oxidation stability over time, ultimately leading to the formation of CuO [29]. This degradation is attributed to AA’s susceptibility to self-decomposition under heat, light, and prolonged storage, which accelerates oxidation of Cu and its oxides [30,31].

To achieve shape-controlled and oxidation-resistant CuNPs, additional capping agents have been investigated, including polyvinylpyrrolidone (PVP) [32], cetyltrimethylammonium bromide (CTAB) [33], sodium dodecyl sulfate (SDS) [34], oleylamine (OA) [35], oleic acid, ethylenediaminetetraacetic acid (EDTA) [36], and ethylenediamine (EDA) [37]. In our work, we explored the role of PVP as both a stabilizer and shape-directing agent, observing a reduction in particle size during synthesis [38]. The pyrrolidinone ring in PVP contributes to its inherent stability, with the amide group resisting hydrolysis and oxidation. Its five-membered cyclic structure and nitrogen moiety further enhance stability [39]. PVP remains stable under alkaline conditions, is only mildly affected by acidic pH [40], and exhibits thermal stability up to 175 °C when supported by metal oxides such as SiO_2_ [41]. In contrast, polymers like polyvinyl alcohol (PVA) and polyethylene glycol (PEG) show lower capping effectiveness due to the absence of non-polar groups necessary for hydrophobic interactions in solvent environments [42].

Among various synthesis methods, microwave-assisted synthesis [43] offers rapid and uniform heating of reactive species, enabling shorter reaction times, improved homogeneity, and controlled nanoparticle morphologies. Given the objective of this study—to achieve oxidation stability at elevated and ambient temperatures over time—the persistent capping of ligands is critical. Previous reports have demonstrated that such capping effectiveness can be maintained during microwave (MW) processing [44,45,46]. Cu particles synthesized via MW methods have been reported to range in size from 3 to 90 nm [47,48,49,50,51,52,53,54,55,56], though discussions on the influence of microwave power and duration remain limited. To complement this approach, we also employed a conventional chemical reduction (CR) method to evaluate whether comparable oxidation resistance could be achieved with fewer synthesis steps and simpler equipment.

To the best of our knowledge, capping agents such as cetyltrimethylammonium bromide (CTAB), ascorbic acid (AA), polyvinylpyrrolidone (PVP), lauric acid, oleylamine, and sodium metaphosphate exhibit oxidation resistance for up to 22 days. As a benchmark, a graphene-like matrix has been shown to prevent Cu oxidation for as long as 60 days. In our previous work, we investigated the capping effectiveness and reaction mechanisms of PVP and AA around Cu particles, demonstrating oxidative stability for up to three months [57]. Regarding thermal oxidation, several studies report that Cu oxidation can occur at temperatures as low as 80 °C—even under vacuum conditions [58]. Additional research has shown that Cu film thicknesses can increase from 40 nm to 100 nm within a week due to oxidation [59]. Thermal and oxidative stability are critical for applications such as steam generation [60] and water desalination [61]. Accordingly, we selected 150 °C as the target temperature for evaluating Cu particle oxidation resistance. Furthermore, we establish protocols for achieving oxidation-stable Cu particles at elevated temperatures (150 °C), highlighting both temporal and thermal stability under ambient conditions without inert atmospheres. We thus conclude that these findings raise the previous benchmarks specifically on oxidation resistance and the preservation of metallic Cu, and distinguish our work from prior reports by combining precursor effects, ligand concentration control, and extended storage stability into a unified framework for practical plasmonic applications. This work demonstrates significant progress in preserving the pristine phase of Cu particles without detectable oxide signatures at 150 °C, opening new avenues for practical applications. A key novelty of this study lies in establishing temporally and thermally stable Cu particles under ambient conditions over extended storage periods—eliminating the need for inert atmospheres. We elucidate the mechanisms underlying the observed oxidation resistance and probable self-limiting behavior by maintaining rigorous consistency in characterization throughout the oxidation analysis. In summary, we present protocols for achieving oxidation-stable Cu particles at elevated temperatures, with broad implications for applications ranging from catalysis to energy storage. Specific examples of such plasmonically stable particles can be envisioned in phase-change-based photothermal energy storage, in catalysis involving reactions above room temperature and plasmon-enhanced catalysis, in temperature-stable optical filters, in high-temperature gas sensing, etc.

## 2. Materials and Methods

Copper nitrate trihydrate (Cu(NO_3_)_2_·3H_2_O—99% purity) and Copper sulphate pentahydrate (Cu(SO_4_)·5H_2_O—99% purity) were procured from Sigma-Aldrich, St. Louis, MO, USA. Polyvinyl pyrrolidone ((C_6_H_9_NO)_n_—99% purity—MW: K30) was obtained from Molychem, Mumbai, India and L-Ascorbic Acid (C_6_H_8_O_6_—99% purity) from Loba Chemie, Mumbai, India. All reagents were used as received without further purification. Deionized water (Vent filter MPK01, St. Louis, MO, USA) was used throughout the experiments. Microwave-assisted reactions were conducted using the Microwave Catalyst 2R model, India, operating within a power range of 85–850 W. The synthesis methodology is divided into two sections: Section 2.1 outlines the distinct synthesis approaches employed (chemical reduction and microwave-assisted), while Section 2.2 details the protocols for conducting stability assessments over time and under thermal conditions, along with the characterization techniques used.

### 2.1. Preparation of Cu Structures

#### 2.1.1. Synthesis Protocol A—Microwave Synthesis

Microwave-assisted synthesis was conducted using a fixed power setting of 680 W for a total duration of 6 min, as established in our previous study which examined the influence of microwave power and ascorbic acid (AA) concentration on particle morphology and size distribution. The selected power level (680 W) yielded a narrower particle size distribution compared to lower power settings.

The synthesis began with preheating 10 mL of the respective copper precursor solution (10 mM) at 680 W for 2 min. Aqueous PVP (0.03–3 mM) was then added to the precursor solution and subjected to microwave irradiation at 680 W for an additional 2 min, during which a visible color change to green was observed. Subsequently, AA was introduced into the mixture, followed by a final microwave irradiation step at 680 W for 2 min. This resulted in the formation of a reddish-brown precipitate, indicating the successful synthesis of Cu particles.

The precipitated particles were collected by centrifugation at 8000 rpm using deionized water and redispersed through three washing cycles to remove residual reactants and stabilize the colloidal suspension.

#### 2.1.2. Synthesis Protocol B—Chemical Reduction

An aqueous solution of either copper nitrate or copper sulphate precursor (10 mM in 10 mL) was mixed with varying concentrations of PVP (0.03 mM, 0.3 mM, or 3 mM) and stirred at 500 rpm for 30 min at room temperature (30 °C). Ascorbic acid (0.5 M) was then added to the mixture, resulting in the formation of a reddish-brown precipitate, indicative of Cu particle synthesis.

The CR protocol mirrors the MW-assisted method in terms of reagent types, concentrations, reaction volume, and addition sequence. The only distinguishing factor is the absence of microwave irradiation, with the reaction proceeding entirely at ambient temperature.

The resulting Cu particles were collected by centrifugation at 8000 rpm in deionized water and redispersed through three washing cycles, identical to the MW protocol. Due to the susceptibility of the synthesized particles to ambient oxidation—whether coated on substrates or stored as powders—all samples were dispersed in deionized water for storage. After twelve months of ambient storage, each sample was subjected to oxidation stability analysis, as detailed in Section 2.2. For clarity and consistency, sample abbreviations are provided in Table 1.

### 2.2. Protocols Followed for Stability Assessment

The oxidation stability of Cu particles was evaluated by monitoring phase and morphological changes before and after thermal exposure. Samples were divided into two groups:

- Group 1: Post-synthesized (PS) samples were tested immediately after synthesis. Each sample (100 μL) was drop-cast onto a 1 cm × 1 cm glass substrate and characterized using X-ray diffraction (XRD) and scanning electron microscopy (SEM). These samples were subjected to thermal exposure at 150 °C for 5 min in a custom high-temperature furnace, with a ramp rate of 10 °C per minute under ambient conditions.
- Group 2: Samples stored in deionized water for twelve months were selected from our previous report [57] specifically, NC2 and SC2. These aged samples were drop-cast and characterized before thermal treatment, followed by identical exposure at 150 °C for 5 min.

This protocol enabled comparative analysis of thermal stability and oxidation resistance across synthesis methods, precursor types, and aging conditions.

**Figure 1:**
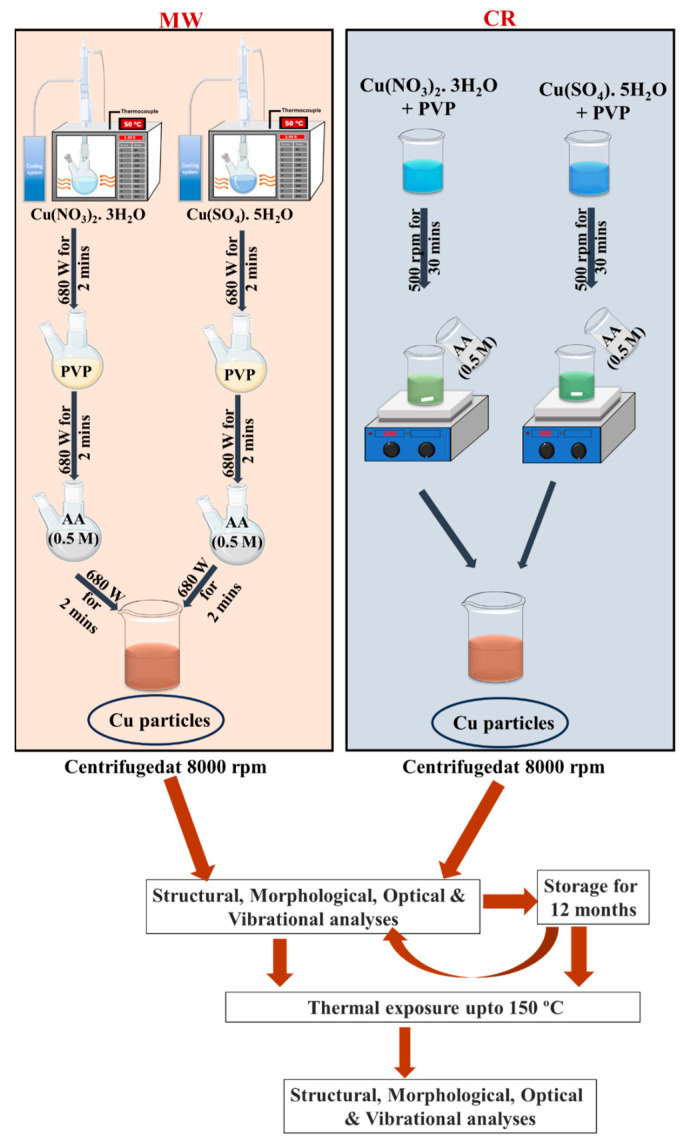
Schematic representation of the sequential protocol for Cu particle synthesis, characterization, and stability evaluation. The workflow outlines the stepwise process beginning with synthesis via chemical reduction (CR) and microwave-assisted (MW) methods using nitrate and sulphate precursors.

The workflow (In Figure 1) illustrates the synthesis routes, chemical reduction (CR) and microwave-assisted (MW), using nitrate and sulphate precursors, followed by characterization and stability testing. Samples were divided into two groups: those assessed immediately post-synthesis and those stored in water for twelve months. Thermal exposure at 150 °C for 5 min was applied to both groups. Acronyms BT and AT denote characterization conducted before thermal exposure and after thermal exposure, respectively.

### 2.3. Characterizations

The Samples were centrifuged at 8000 rpm for 10 min using a REMI R-24 centrifuge with an RCF value of 27,440 g before being subjected to characterization. The samples’ phase purity and crystallinity were investigated by X-ray diffraction (XRD Empyrean, Malvern Panalytical, Westborough, MA, USA) with a Cu-Kα source (λ = 1.54 Å) and the diffractograms recorded between 2θ angles of 20° and 80° with instrumental broadening correction built-in. The phase and structural corroborations were performed with a Crystallographic Open Database (COD) using X’Pert High Score Plus. The fabricated samples were optically characterized with UV-Vis absorbance spectroscopy with achromatic light from a tungsten halogen lamp (HL-2000 from Ocean Optics, Orlando, FL, USA, 300 nm to 2000 nm) passing through the sample with a path length of 1 cm. An optical fiber cable was connected from the collimating lens to the spectrometer (Ocean Optics Flame-T-Vis-NIR) to obtain the spectra. Binding interactions were analyzed by Fourier Transform Infrared (FTIR, Jasco 4100, Tokyo, Japan) spectroscopy using a double beam spectrometer wherein transmittance was collected between 4000 cm^−1^ and 550 cm^−1^ in Attenuated Total Reflection (ATR) mode. Morphological images were obtained using Scanning electron microscopy (SEM—ZEISS EVO 18, Oberkochen, Germany) with an operating voltage of 20 kV. The zeta potential was optically analyzed by Dynamic Light Scattering (Malvern Panalytical-Zetasizer version 7.13, Westborough, USA).

## 3. Results

The morphologies, surface potentials, optical properties, crystallinity, and nature of capping molecules were investigated along three primary directions. Section 3.1 details the choice of reagents like precursors and their concentrations. Section 3.2 focuses on the influence of synthesis method-dependent variations on the structural, morphological, vibrational, and optical characteristics of the Cu particles. Section 3.3 examines how these same properties evolve in response to thermal exposure and prolonged storage in an aqueous medium. Finally, Section 3.4 presents a comparative analysis, correlating the observed results with existing literature to contextualize and validate the findings.

### 3.1. Choice of Reagents and Concentrations

An identical precursor concentration of 10 mM was selected for both copper nitrate and copper sulphate salts to ensure consistency across synthesis routes. The reducing agent concentration was fixed at 0.5 M, based on our previous report [57], to promote rapid nucleation and minimize particle size. Polyvinyl pyrrolidone (PVP) concentrations were varied across three levels: 0.03 mM, 0.3 mM, and 3 mM. The intermediate concentration (0.3 mM) was previously shown to yield oxidation-stable particles for up to three months in aqueous storage. The lower (0.03 mM) and higher (3 mM) concentrations were chosen to investigate the influence of PVP content on particle dispersity, surface passivation, and morphology. Notably, anisotropic structures have been reported at PVP concentrations approaching these values [62,63,64,65], supporting their inclusion in this study. The chosen precursors of copper were copper sulphate and copper nitrate, whose differences in solubility and the reduction rate, for example, can be explained with the Hofmeister series, which identifies ${SO}_{4}^{2-}$ as a chaotropic anion and ${NO}_{3}^{-}\;$ as a kosmotropic anion [66,67,68] as follows

(1) $${SO}_{4}^{2-}>{Cl}^{-}>{NO}_{3}^{-}$$

The ionic bond between Cu^2+^ and ${SO}_{4}^{2-}$ is more stable than that between Cu^2+^ and ${NO}_{3}^{-}$ due to the equal sharing of ions in the former. The solubility of copper nitrate is thus higher, which influences the reduction in the precursor particle properties as per the Lamer model [69], which proposes that nanoparticle formation starts with nucleation (termed burst nucleation, also known as instantaneous nucleation) [70] and proceeds to particle growth dependent on temperature and concentration of the precursor atoms. Copper sulphate also has a higher charge density than other examples, such as nitrate, chloride, and acetate precursors.

The expected reactions of AA and PVP with Copper precursors are as follows (R1):


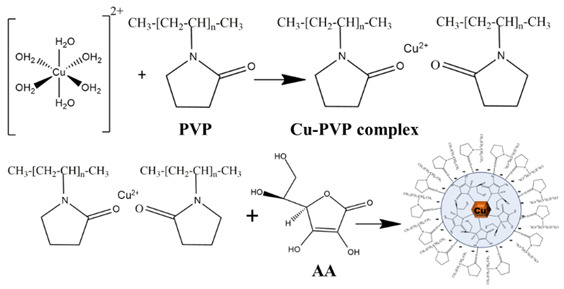


During the synthesis process, polyvinylpyrrolidone (PVP) initially complexes with Cu^2+^ ions through coordination at the oxygen atoms of its polar groups, forming stable metal–O_2_ interactions. Subsequent addition of ascorbic acid (AA) results in electron donation via homolytic cleavage of an OH bond. The carbonyl double bond within the lactone ring of AA conjugates with Cu^+^, producing a characteristic green coloration. AA is then oxidized to dehydroascorbic acid, while Cu^+^ undergoes complete reduction to metallic Cu^0^, accompanied by a color change to brown. The residual positive charge of PVP is proposed to remain associated with the particle surface, contributing to the observed surface charge.

### 3.2. Discussions on Synthesis Method-Based Variations

#### 3.2.1. Morphological Analyses

**Figure 2:**
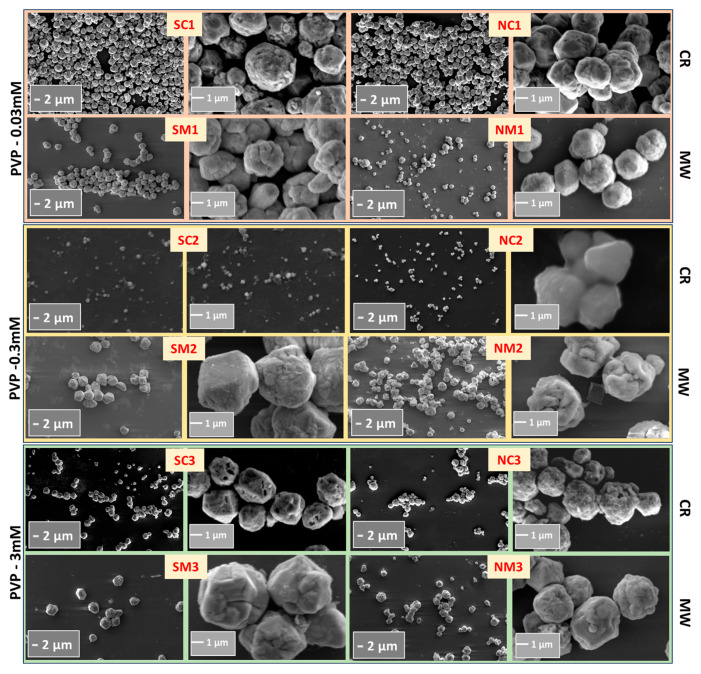
Scanning electron micrographs of Cu structures synthesized via microwave-assisted (MW) and chemical reduction (CR) methods. Images captured at magnifications of 5 KX and 30 KX reveal distinct morphological features influenced by synthesis route.

Figure 2 indicates that the synthesized particles were typically in the micrometer range. In general, chemically reduced (CR) samples exhibited smaller particle sizes compared to those produced via microwave-assisted (MW) synthesis. This disparity may be attributed to a two-step nucleation and growth mechanism inherent to the MW process: the initial addition of ascorbic acid (AA) serves as the nucleation phase, followed by MW irradiation that promotes particle growth through Ostwald ripening and/or secondary nucleation [71]. This two-step behavior stems from the relatively weak reducing nature of AA [72]. MW-assisted samples also displayed a higher degree of faceted growth, suggesting distinct crystallographic plane growth kinetics.

The broad particle size distributions observed in Figure S1 may result from multiple factors, including structural transitions in PVP such as thermal degradation into monomers, which can initiate around 100 °C during MW processing [73] leading to variable steric hindrance. Additional contributors include pH-induced variations, differences in microwave power, reaction duration, solvent systems, precursor types, and concentrations of AA or PVP.

#### 3.2.2. Lattice-Level Visualizations

**Figure 3:**
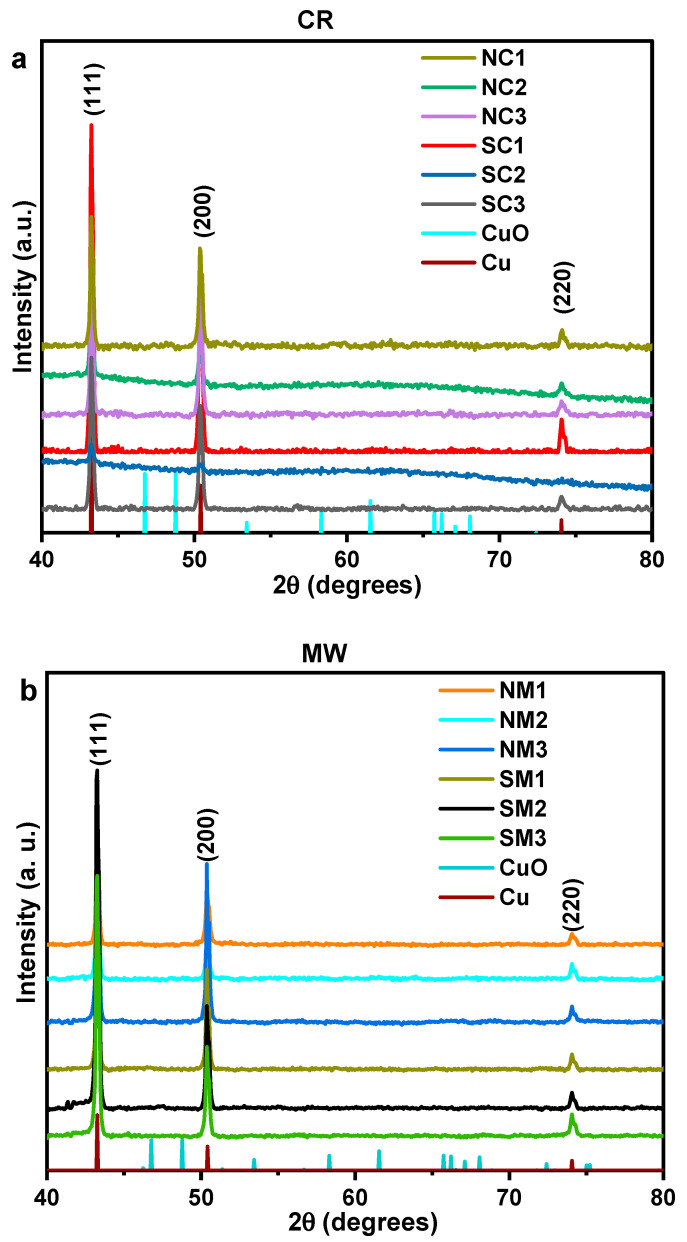
Comparative XRD patterns of Cu particles synthesized from nitrate and sulphate precursors immediately after synthesis. Panel (**a**) shows chemically reduced (CR) samples, while panel (**b**) displays microwave-assisted (MW) samples.

X-ray diffraction (XRD) analysis revealed no detectable oxide peaks, indicating the preservation of the pristine metallic phase—a significant outcome. The diffraction pattern corresponds to a face-centered cubic (FCC) crystal system, with reflections indexed to the (111), (200), and (220) planes, consistent with ICSD No. 53756. The structure is associated with the Fm-3m space group and a unit cell volume of 47.24 × 10^6^ pm^3^, as illustrated in Figure 3. Relative intensities, normalized to the dominant (111) plane, are presented in Supplementary Figure S2. These data suggest a preferential growth along the low-energy (200) plane over the (220) plane, likely due to the chemical reduction (CR) process being conducted at room temperature. Crystallite sizes were calculated using the Scherrer equation, based on an X-ray wavelength of 1.54 Å, and are summarized in Table 2.

#### 3.2.3. Optical Analyses

**Figure 4:**
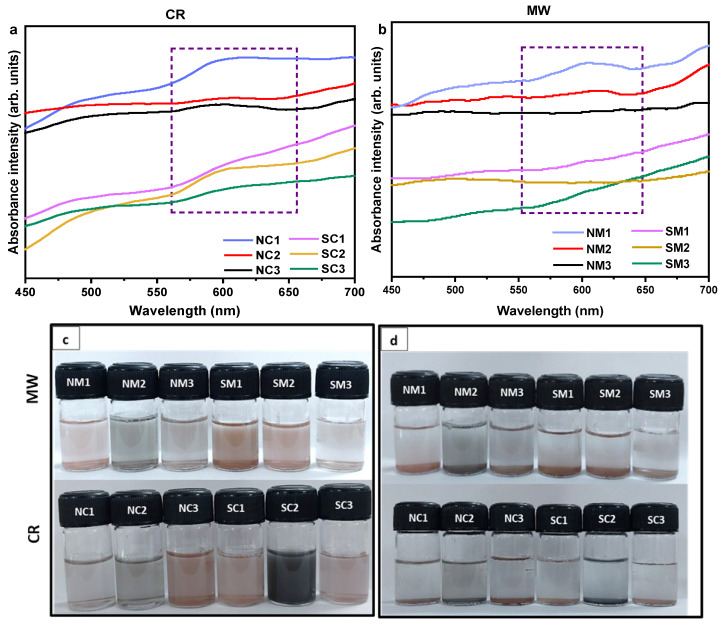
UV-Vis spectra and optical images of Cu particles. Panels (**a**,**b**) show the UV-Vis absorption profiles of samples synthesized via chemical reduction (CR) and microwave (MW) methods, respectively. Panels (**c**,**d**) display optical images of the samples right after and five minutes after sonication, respectively, illustrating dispersion behavior and visual changes in colloidal stability.

Figure 4 presents the absorption spectra of the synthesized copper particles. Samples derived from the nitrate precursor exhibit a more pronounced plasmonic absorbance near 600 nm compared to those synthesized using the sulphate precursor. The absence of a well-defined or symmetric plasmonic peak in certain samples may be attributed to particle aggregation and/or faceted surface morphologies. Additionally, a higher degree of polydispersity in particle sizes can contribute to broader and asymmetric absorbance profiles [74].

Examination of the supernatant, as shown in Figure S3, revealed no absorbance near 600 nm, but a distinct feature was observed in the interband transition region around 350 nm. This suggests that the particles present in the supernatant are non-plasmonic and likely below the size threshold required to support localized surface plasmon resonance.

#### 3.2.4. Vibrational Analyses

**Figure 5:**
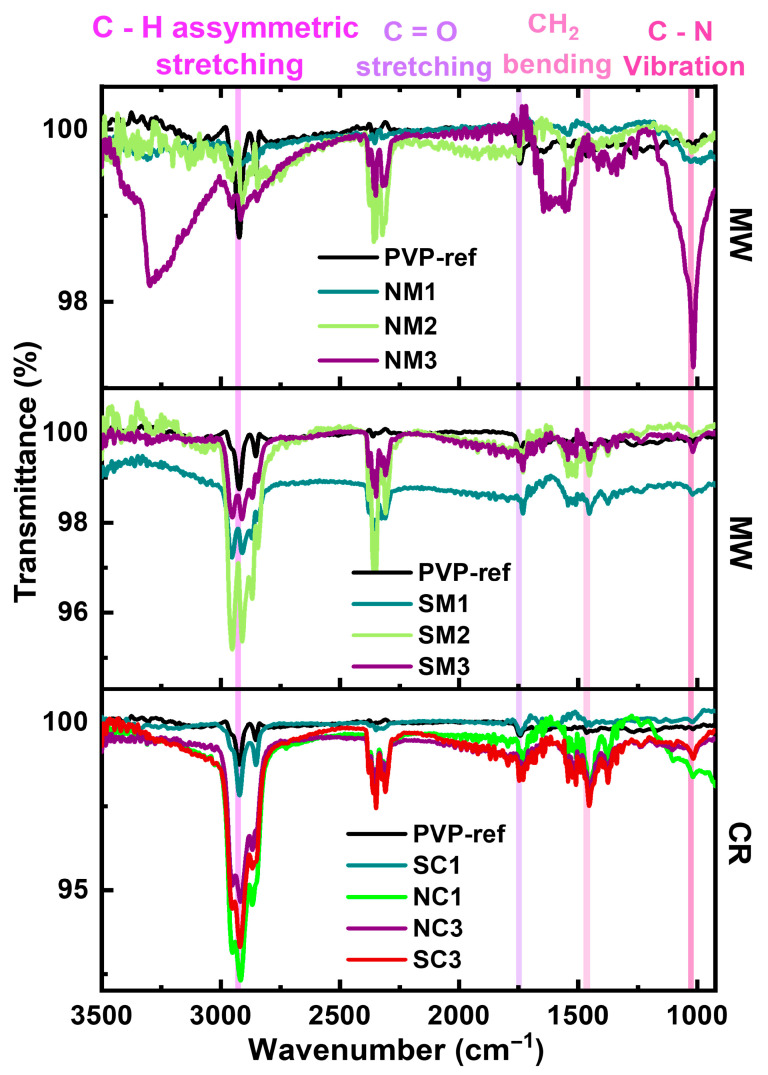
Comparative FTIR spectra of Cu particles synthesized via chemical reduction (CR) and microwave (MW) methods using nitrate and sulphate precursors.

Fourier-transform infrared (FTIR) spectroscopy was employed to evaluate the effectiveness of PVP capping, as shown in Figure 5. Characteristic peaks corresponding to the carbonyl and hydroxyl functional groups of PVP were observed at 1743 cm^−1^ and 3305 cm^−1^, respectively. Additional features include asymmetric C–H stretching and CH_2_ vibrations at 2925 cm^−1^ and 1459 cm^−1^. Furthermore, a distinct peak at 1021 cm^−1^ was attributed to C–N vibrations arising from the pyrrolidone ring structure, confirming the presence and interaction of PVP with the copper particle surface.

#### 3.2.5. Electrokinetic Analysis

**Figure 6:**
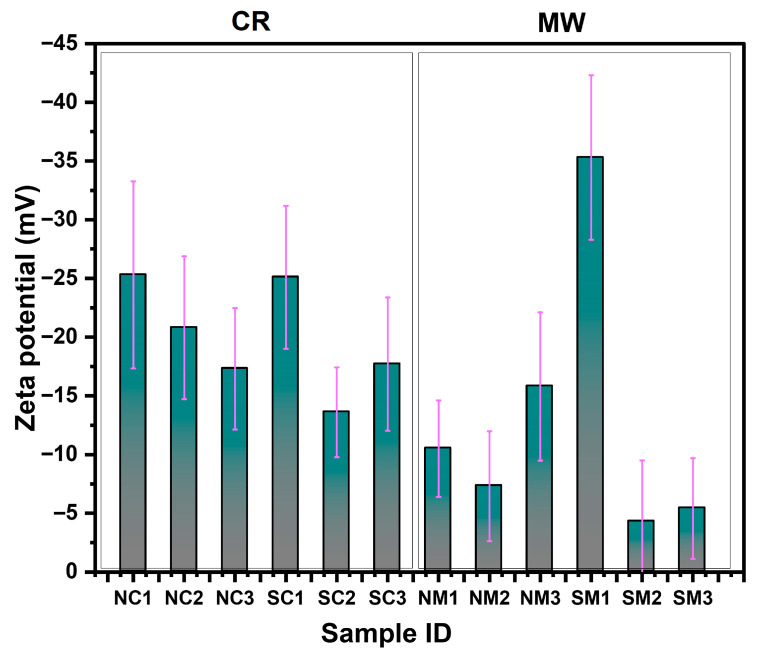
Surface charge (zeta potential) analysis of Cu particles synthesized via chemical reduction (CR) and microwave (MW) methods using nitrate and sulphate precursors. The purple line denotes the standard deviation associated with the zeta potential measurements of each sample.

Surface charge analysis was conducted using dynamic light scattering (DLS) following a 2 min sonication. The zeta potential values for the NM2, SM2, and SM3 samples shifted toward a more positive range compared to the other samples, suggesting a relatively weak capping strength of PVP and AA for these samples. As shown in Figure 6, an inverse relationship between particle size and zeta potential was observed samples with larger particle sizes exhibited lower zeta potential values. This highlights the influence of particle morphology and surface chemistry on colloidal stability.

The NC2 and SC2 samples exhibited zeta potential values < −10 mV, indicating a sustained capping effect even after twelve months of storage in aqueous solution. The analyses conducted thus far have provided valuable insights into the capping effectiveness, dispersion behavior, oxidation susceptibility, optical characteristics, morphological features, surface properties, and crystallinity including lattice development of the synthesized Cu particles. Building on these findings, the next section will explore the effects of ambient storage and thermal treatments on the stability and structural integrity of the capped Cu particles.

### 3.3. Investigations on Sample Variations Post Time/Thermal Exposures

We now turn our attention to the temporal and thermal evolution of the synthesized copper particles. Section 3.3.1 examines changes in particle characteristics following prolonged storage in aqueous media under ambient conditions, while Section 3.3.2 explores the effects of elevated temperature exposure. Both analyses focus on the particles’ propensity toward oxidation, providing insight into their long-term stability and suitability for practical applications.

#### 3.3.1. Influence of Ambient Storage

**Figure 7:**
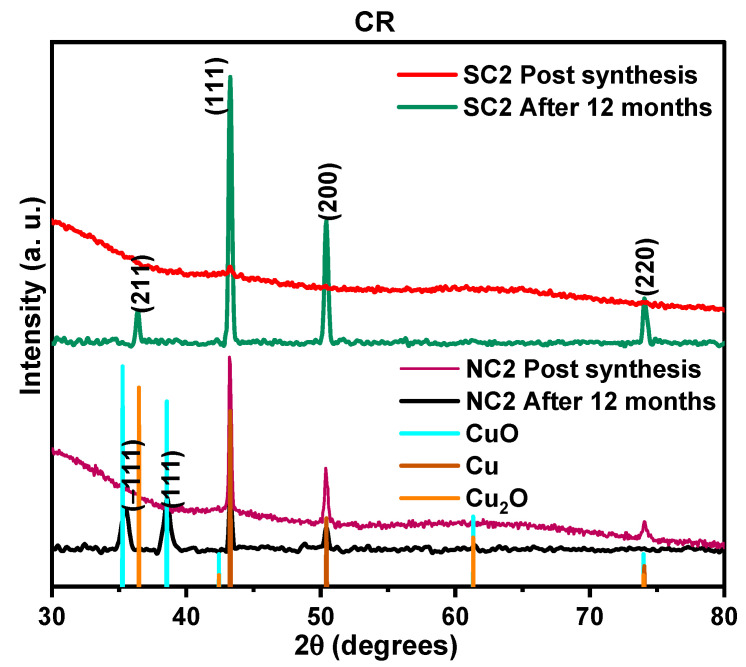
XRD patterns of chemically reduced (CR) Cu particle samples immediately after synthesis and after twelve months of storage.

For the ambient storage analysis, the procedure involved: (i) storing the Cu particle samples in deionized water, and (ii) after 12 months, drop-casting them onto three different substrates one designated for thermal treatment studies and the remaining two for characterization. X-ray diffraction (XRD) results for the SC2 sample revealed that the characteristic Cu diffraction peaks at 43.38°, 50.52°, and 74.24° remained unchanged after 12 months, as shown in Figure 7, indicating structural stability. Notably, Cu_2_O was detected in the sample synthesized using the sulphate precursor, while CuO was observed in the nitrate-derived sample, suggesting a more advanced oxidation state in the NC2 sample. These findings underscore the significant influence of precursor chemistry on the oxidative behavior of copper particles. Polyvinylpyrrolidone (PVP) appears to permit oxygen diffusion to the copper surface, allowing oxidation to occur despite its capping role. It is possible that the resulting oxide layer probably exhibits self-limiting behavior [75]. Within the PVP structure, coordination between nitrogen atoms and metallic copper contributes to stabilization against oxidation; however, this interaction is evidently insufficient for complete protection, as oxidation still progresses over time.

**Figure 8:**
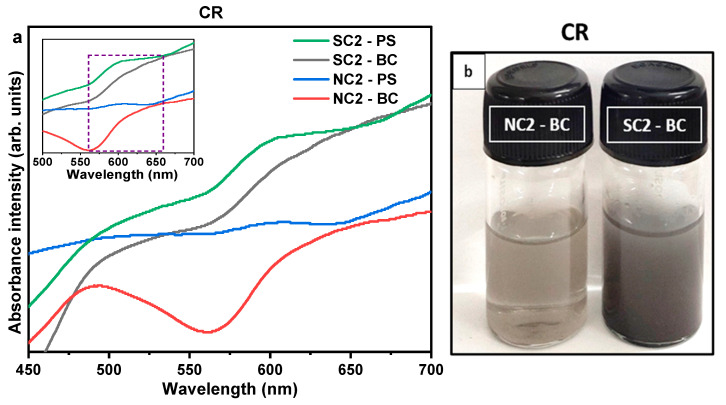
UV-Vis spectra of NC2 and SC2 samples post-synthesis (PS) and after twelve months (BT). (**a**) The main plots show spectral changes over time, with notable shifts in absorption features indicating surface oxidation and ligand degradation. Insets display magnified regions from 500 nm to 700 nm, highlighting the loss or broadening of surface plasmon resonance (SPR) peaks. (**b**) presents the spectra of NC2 and SC2 after twelve months, emphasizing the long-term optical evolution of the samples.

As shown in Table 3, the average crystallite size decreased from the post-synthesis stage to post-ambient storage, likely due to the growth of oxide phases. Corresponding changes in absorbance are presented in Figure 8. The oxidation process appears to be governed by a complex interplay of lattice strain induced by oxygen incorporation, structural evolution of oxide phases through grain growth, and defect formation such as cracks and voids. This multifaceted behavior complicates a complete mechanistic understanding of the oxidation pathway. Notably, scanning electron microscopy (SEM) images of the NC2 and SC2 samples did not reveal hollowing an expected outcome of the Kirkendall effect, which involves differential diffusion rates of metal and oxygen atoms. This absence suggests that rather than full oxidation, a reconstruction of Cu_2_O and CuO phases around a metallic Cu core may have occurred.

The absorption peaks for the NC2 and SC2 samples exhibited noticeable broadening, likely resulting from particle aggregation and oxidative shell formation, which alter the local refractive index [76]. Such changes can induce a redshift in the plasmonic resonance, as predicted by the plasmonic frequency equation [77]. Interband transitions from the Fermi surface to higher unoccupied energy levels were observed at 492 nm for NC2 and 500 nm for SC2 [78]. Metallic copper presence is confirmed in Figure 8 by the broad absorption feature spanning 600–750 nm, although the peak is more dampened in NC2 than in SC2, indicating a higher degree of oxidation. Additionally, the visible color change in both samples from brown to dark brown further supports the observed spectral shifts, consistent with oxidation and agglomeration effects [57]. According to Mie’s solution for light scattering, an increase in the dielectric constant of the surrounding medium leads to a redshift in the absorbance wavelength [77].

(2) $$Q_{ext}=\frac{24\pi a}{\lambda }\frac{\varepsilon_{2}\varepsilon_{m}}{{{(\varepsilon }_{1}+2\varepsilon_{m})}^{2}+{\varepsilon_{2}}^{2}}$$

where a and $\varepsilon_{m}$ are the particle size and dielectric constant of the surrounding medium, respectively. $\varepsilon_{1}$ and $\varepsilon_{2}$ are the real and imaginary parts of the material dielectric function.

Vibrational analysis was employed to assess structural changes in the pristine Cu phase of the NC2 and SC2 samples following ambient storage. The spectral features confirmed modifications in the interaction between PVP and the copper surface, which likely contributed to the oxidation signatures observed in earlier analyses. 

**Figure 9:**
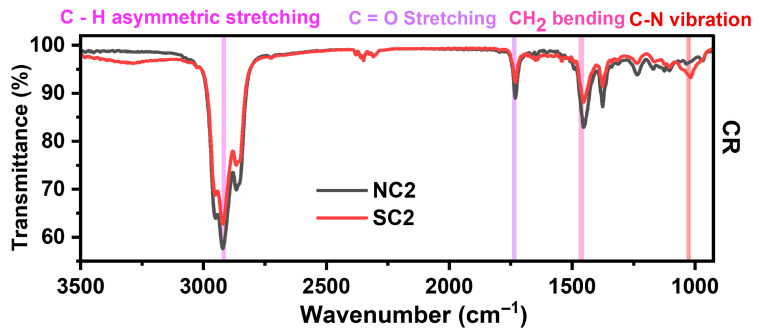
FTIR spectra of NC2 and SC2 samples after twelve months of storage.

Despite prolonged ambient storage, the functional groups of PVP remain detectable, as supported by previous reports [38,79]. This suggests that PVP continues to play a role in modulating the rate of copper oxidation. The FTIR peak at 1732 cm^−1^, indicative of vibrational interactions between the carbonyl (C=O) group and the Cu metal surface, warrants particular attention. As the primary functional group of PVP, C=O typically coordinates with metal ions during capping, which can weaken over time. However, the pronounced intensity of this peak implies a sustained and effective capping interaction with metallic Cu^0^. Additionally, the peak at 1377 cm^−1^ corresponds to the enol-hydroxyl group of ascorbic acid (AA), represented by the structure –C(OH)=C(OH)–C(=O)–, as shown in Figure 9. These observations indicate that both PVP and AA contribute to the oxidative kinetics of the samples, influencing their stability and transformation pathways.

**Figure 10:**
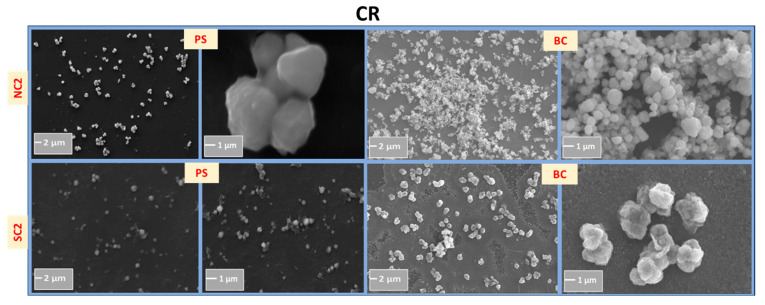
Comparative SEM images of NC2 and SC2 samples immediately after synthesis and after twelve months of storage.

The average particle size for the NC2 sample increased from 0.85 μm ± 0.1 to 1.64 μm ± 0.4, and for SC2 from 0.35 μm ± 0.1 to 0.54 μm ± 0.1, as shown in Figure 10. This growth may be attributed to oxidation or particle aggregation. No evidence of anisotropic growth was observed. Aggregation and agglomeration in solution are influenced by factors such as pH, ionic strength, particle mobility, and solvent viscosity. A higher degree of aggregation is likely when particles are suspended in an aqueous medium compared to when they are immobilized on a substrate.

These findings from lattice-level visualizations to particle-level properties highlight key aspects of copper particle behavior, including oxidation tendency, the role of PVP in modulating oxidation, aggregation dynamics, and the impact of solvent-based storage. Building on these insights, the next section investigates the stability of the samples when subjected to elevated temperatures (150 °C), with a focus on their resistance to oxidation.

#### 3.3.2. Influences of Thermal Exposures

**Figure 11:**
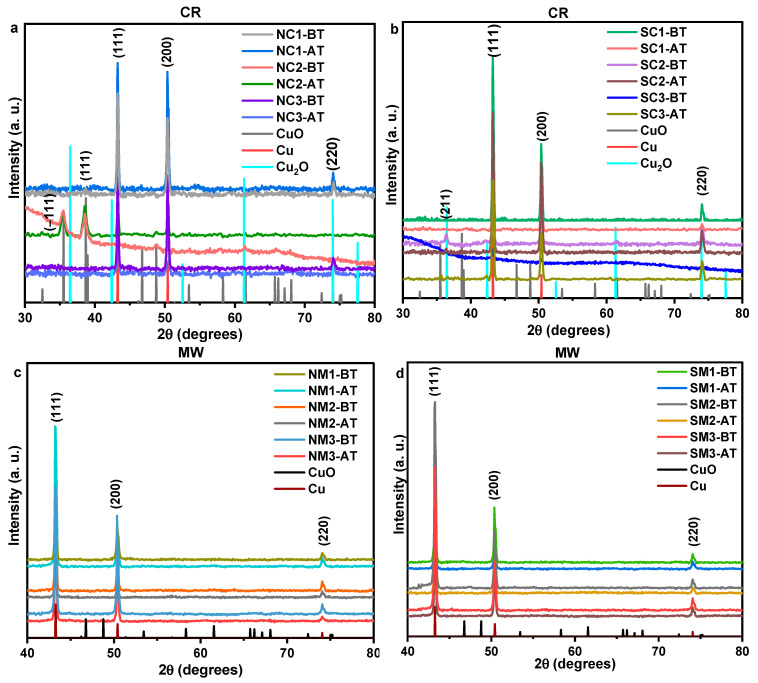
XRD of CR and MW synthesized nitrate precursor (**a**,**c**) and sulphate precursor (**b**,**d**) samples before and after the thermal exposure.

X-ray diffraction (XRD) analysis of the group 1 samples revealed no evidence of oxide phase formation, as indicated by the absence of characteristic oxide diffraction planes. Interestingly, for group 2 samples, the diffraction planes associated with oxide phases such as (−111), (111), and (211)—remained unchanged, with no observable shifts in phase proportions, emergence of new planes, or transitions between oxide phases, as shown in Figure 11. These findings suggest the presence of either a self-limiting or slow-growing oxide phase that effectively restricts further oxygen diffusion into the metallic core, thereby stabilizing the crystallinity of the copper particles. Such behavior has been previously reported [80] and supports the hypothesis of a passivating surface layer that mitigates progressive oxidation.

Crystallinity parameters are summarized in Table S1, with values rounded to the second decimal place any changes beyond this precision were assigned a value of zero. Following thermal exposure, a decrease in crystallite size was observed across nearly all samples. This reduction may be attributed to grain refinement induced by thermal treatment, as well as an increase in dislocation density [81]. Notably, group 2 samples did not exhibit complete oxidation even at elevated temperatures, underscoring their thermal resilience.

**Figure 12:**
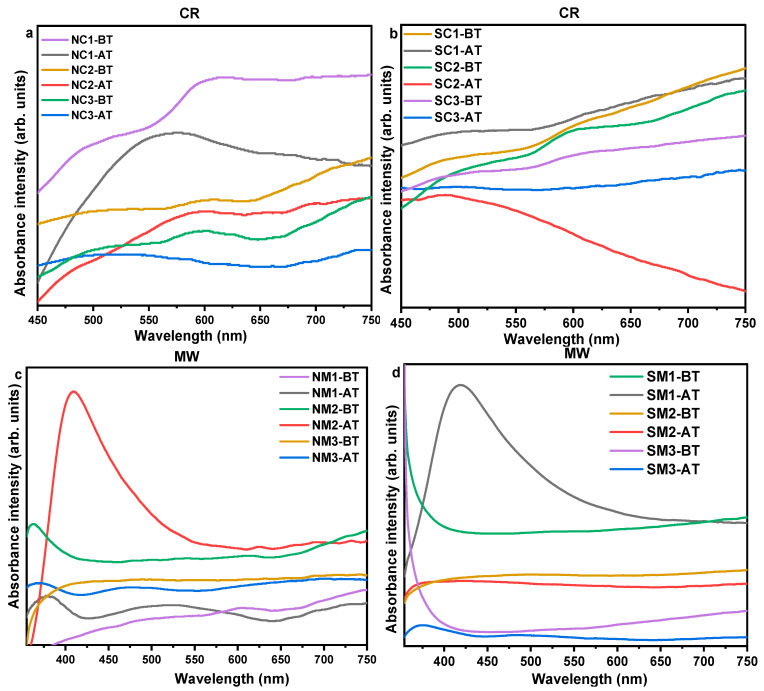
Comparative UV-Vis spectra of Cu particles synthesized via chemical reduction (CR) and microwave (MW) methods before and after thermal exposure. Panels (**a**,**c**) correspond to samples synthesized using nitrate precursors, while panels (**b**,**d**) represent those from sulphate precursors. The spectra reveal changes in surface plasmon resonance (SPR) and interband transitions, reflecting the influence of precursor type and synthesis method on optical properties and thermal stability.

Relative intensities of the diffraction planes were calculated and normalized to the (111) plane, as shown in Figure S4, revealing shifts in phase proportions. UV-Vis spectral analysis post-thermal exposure (Figure 12) showed that most samples except SC3, SC1, NM1, NM3, SM2, and SM3 lacked a discernible surface plasmon resonance (SPR) peak in the visible region. Some samples, such as NM2 and SM1, exhibited interband transitions around 420 nm and 360 nm, respectively. Additional transitions were observed at 384 nm (SC3) and 396 nm (SC2), corresponding to electronic transitions from d to sp bands [78].

**Figure 13:**
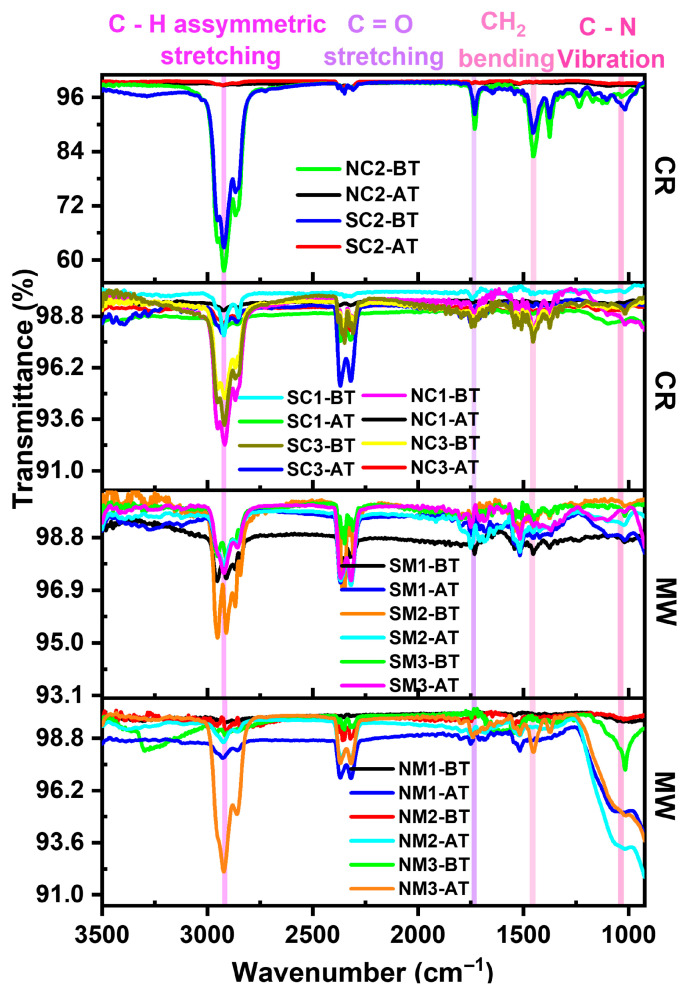
Comparative FTIR spectra of Cu particles synthesized via chemical reduction (CR) and microwave (MW) methods using different precursors.

FTIR analysis of thermally treated samples was compared with spectra obtained immediately after synthesis to evaluate the temperature-dependent capping efficiency of PVP. The carbonyl (C=O) stretching vibration, a characteristic peak of PVP, was consistently observed at 1741 cm^−1^, as shown in Figure 13. The persistence of this peak after thermal exposure suggests that PVP retains its structural integrity and continues to interact with the copper surface, contributing to the stabilization of the particles against oxidation.

Thermal exposure led to the disappearance of OH stretching bands in the FTIR spectra, indicating the weakening of hydroxyl groups. Shifts toward lower wavenumbers may be attributed to diminished molecular interactions at elevated temperatures [73] or to changes in the relative contributions of overlapping vibrational bands commonly referred to as real band frequency shifts. These observations suggest that while PVP has undergone thermal degradation, it has not been entirely eliminated, as evidenced by the persistence of key functional group signals. FTIR data for all samples are compiled in Table S2.

**Figure 14:**
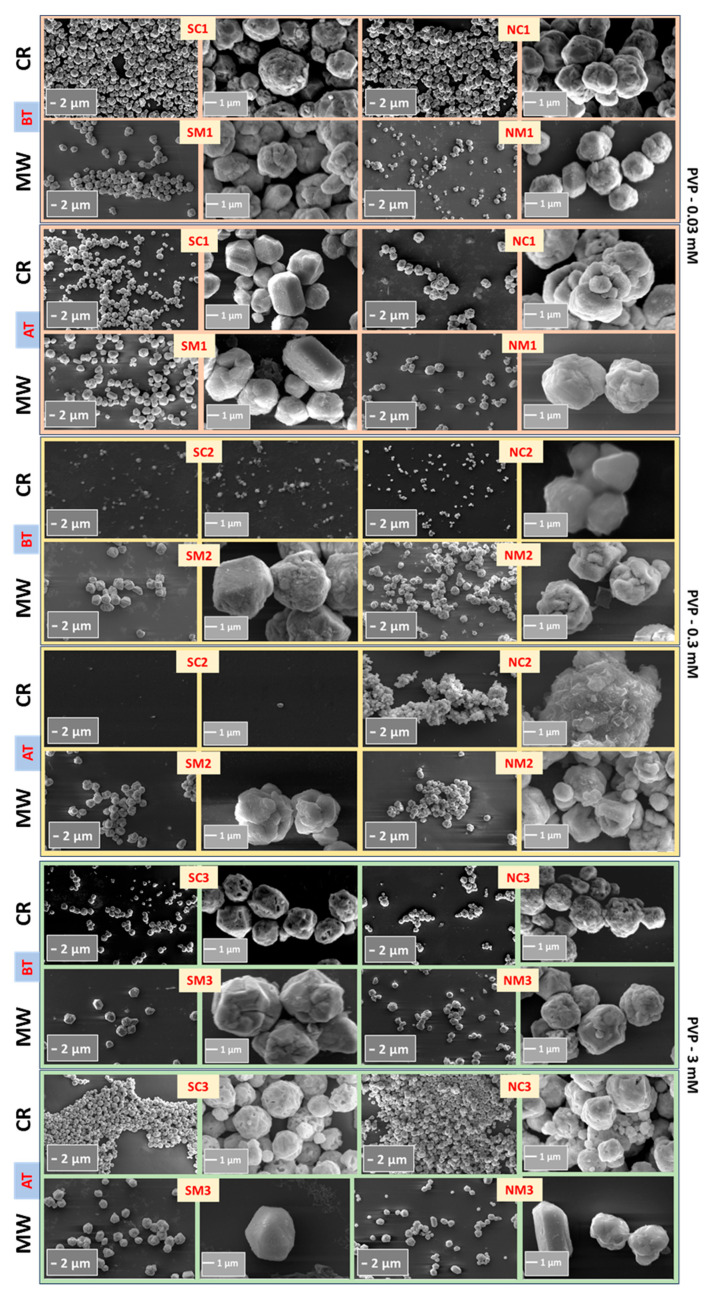
Comparative SEM images of Cu particles before and after thermal exposures.

To assess morphological changes, scanning electron microscopy (SEM) was performed, as shown in Figure 14, with particle size calculations provided in Figure S5. Although the high standard deviation in size measurements limits definitive conclusions, a consistent trend of increased average particle size was observed, likely due to aggregation or agglomeration induced by thermal treatment.

The findings presented in this study make a meaningful contribution to the broader body of research on the oxidative stability and colloidal stability of copper particles. A comparative analysis with existing literature can elucidate the specific enhancements and limitations introduced by the synthesis and treatment protocols employed herein. Furthermore, several unique observations particularly those related to precursor influence, ligand behavior, and morphological evolution offer valuable insights that may inform predictive strategies for tailored Cu particle synthesis. These aspects will be explored in the subsequent section.

### 3.4. An Integrated Physicochemical and Stability Analysis

**Figure 15:**
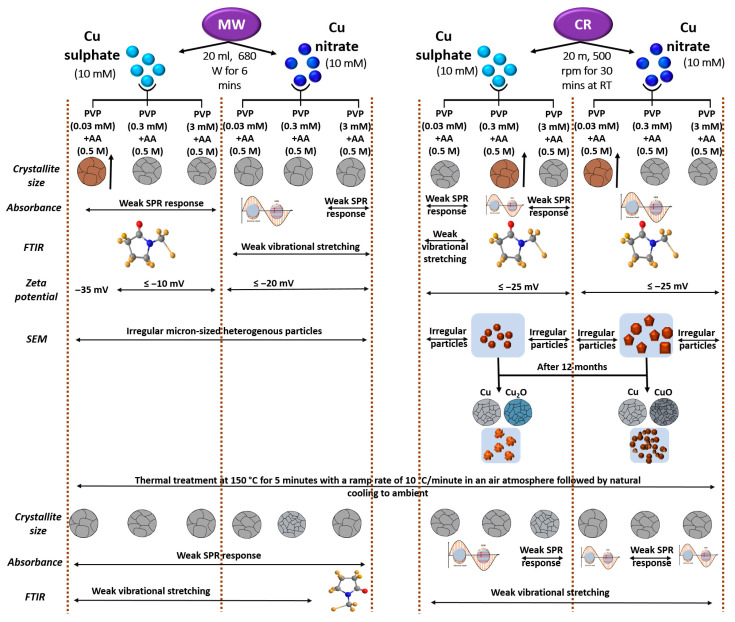
A Schematic representation of Cu particle synthesis via MW and CR methods and their influence on structural, optical, and morphological properties.

A summary overview of this report in the context of the synthesis methods and testing protocols has been presented in Figure 15. It begins with the quantitative synthesis parameters and justification of the precursors, reducing agents, and capping agents employed for the controlled synthesis of Cu particles. Concentration ratios, reaction temperature, reaction time, and atmosphere are included to be able to visualize the conditions required for producing stable and well-defined particles. Key physicochemical and plasmonic properties of the synthesized Cu particles encompassing crystallite size, functional group signatures, surface charge behavior, and particle size distribution as a function of the synthesis conditions have been portrayed. We also illustrate the thermal and plasmonic stability of the samples following the thermal treatment at 150 °C, highlighting structural integrity, potential oxidation effects, modifications in surface chemistry, and shifts in plasmonic resonance.

### 3.5. Comparative Studies on Pristine Cu and Integrated Cu Phases with Published Research

In this study, polyvinylpyrrolidone (PVP) demonstrated partial efficacy as an oxidation retardant. While it allowed oxidation to proceed in the aqueous phase, it provided exceptional protection against thermal-induced oxidation. Evidence of storage-related oxidation was observed through the emergence of weak secondary phases: tenorite (CuO) at 2θ = 36.49° for the SC2 sample, and cuprite (Cu_2_O) at 2θ = 35.43° and 38.73° for NC2. These findings suggest that while PVP offers some stabilization, its effectiveness is context-dependent. Table 4 presents a comparative overview of previously reported copper particle oxidation stabilities under varied storage conditions, highlighting the nuanced role of capping agents in long-term Cu particle preservation.

The outcomes of this work have been highlighted in the comparative table for the clarity of understanding. This comprehensive study positions organic capping agents specifically PVP and AA—as promising alternatives to matrix-like graphene, which, while effective in preventing oxidation, tends to suppress plasmonic behavior. By anchoring these ligands to the stern layer of copper particles, our approach circumvents this suppression, enabling retention of optical activity and opening new avenues for plasmonic applications.

We present a straightforward, solution-based synthesis route for oxidation-stable copper particles that remain largely unaffected by external factors, with storage conditions being the primary variable influencing stability. The data reveal a strong correlation between the choice of capping ligand and long-term particle integrity, attributed to robust surface binding and effective steric or electrostatic repulsion. Additionally, both the synthesis environment and post-synthesis treatments are shown to be critical in preserving plasmonic properties and mitigating degradation.

The synergistic action of AA and PVP provides notable thermal stability for both oxidized (NC2 and SC2) and metallic Cu phases. However, despite their protective roles, these agents do not fully prevent gradual oxidation over time, as evidenced by minor oxide phase signatures. The underlying mechanisms of this oxidation remain under investigation.

Overall, the chemical compositional stability achieved through this method enhances the viability of copper particles for diverse applications, including conductive inks, catalysis, and optoelectronic devices.

## 4. Observational Summary

**Figure 16:**
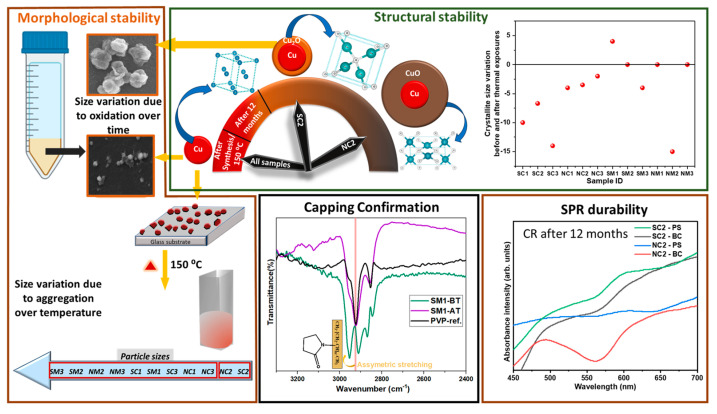
Illustrative summary of Cu particle stability under varying conditions.

For a more concise report on the findings of this study, Figure 16 has been illustrated to highlight the findings. We emphasize the aspects of differences in morphological stability and morphology, that of oxidation resistance and plasmonic retention of crystallinity changes and phase changes. We also highlight the precursor-dependent phase changes in the samples, the important investigation of a time-observed oxidation behavior and equally importantly, its comparison with temperature-dependent oxidation behavior. We also emphasize the stabilizing nature of the chosen reagents, which has enabled this stability against oxidation to be imparted to the Cu particles.

## 5. Conclusions

Cu particles synthesized via solution-based methods showed oxidative stability up to 150 °C, with XRD confirming retention of the pristine Cu phase. When comparing the samples before and after thermal treatment, the crystallite sizes of the pristine Cu phase were consistently measured at approximately 38 nm for the majority of samples. This value suggests that the copper phase retains its structural integrity under the applied conditions. Storage in aqueous media led to Cu_2_O formation from sulphate precursors and CuO from nitrate precursors after 12 months stored at ambient conditions. This study hence demonstrates a solution-based synthesis of copper nanoparticles with enhanced oxidative stability, primarily influenced by storage conditions rather than thermal exposure. XRD confirmed the preservation of the pristine Cu phase after heating to 150 °C, provided the particles were not dispersed long-term in aqueous media. Storage-induced oxidation was precursor-dependent, with Cu_2_O forming from sulphate and CuO from nitrate sources. Notably, thermal treatment did not exacerbate oxidation, suggesting an oxide phase formation that is interestingly inert to this treatment. Previous investigations under vacuum conditions revealed oxidation occurring at 80 °C. By contrast, the reagent combination employed in this study has reduced the extent of oxidation.

The combined use of Cu nitrate/sulphate (10 mM): PVP (0.003, 0.03, 0.3 mM): AA (0.5 M) in the ratios of 1:0.0003/0.003/0.03:50 provided thermal stability and partial protection against aqueous oxidation. FTIR and UV-Vis analyses supported the retention of functional groups such as CH asymmetric stretching and plasmonic features, though minor oxide signatures emerged over time. These findings highlight the critical role of ligand chemistry and post-synthesis handling in preserving copper’s structural and optical integrity, offering a practical route for applications in conductive inks, catalysis, and optoelectronics.

## Figures and Tables

**Table 1:** Abbreviated sample names for the samples from different concentrations of PVP and the precursors.

PVP Concentrations	Samples from the Nitrate Precursor		Samples from the Sulphate Precursor	
	CR *	MW *	CR	MW
0.03 mM	* NC1	* NM1	* SC1	* SM1
0.3 mM	NC2	NM2	SC2	SM2
3 mM	NC3	NM3	SC3	SM3

* CR—Chemical reduction, MW—Microwave, N—nitrate, S—Sulphate, C-chemically reduced, M-microwave synthesized.

**Table 2:** Comparative crystallite sizes of Cu particles synthesized via microwave-assisted (MW) and chemical reduction (CR) methods.

	CR						MW					
Samples	NC1	SC1	NC2	SC2	NC3	SC3	NM1	SM1	NM2	SM2	NM3	SM3
Crystallite size (nm)	39.89	36.56	37.38	39.38	36.56	36.56	38.15	39.89	38.15	36.56	38.15	36.56

**Table 3:** Average crystallite sizes of different Cu phases in particles subjected to ambient storage.

Sample Name		hkl	Crystal Size (nm)	Average Crystal Size (nm)
**NC2 (Post synthesis)**	Cu	(111)	37.38	36.86
		(200)	33.25	
		(220)	39.96	
**NC2 (Post 12 months)**	CuO	(−111)	13.79	14.48
		(111)	15.15	
	Cu	(111)	25.04	24.35
		(200)	23.64	
**SC2 (Post synthesis)**	Cu	(111)	39.38	35.86
		(200)	32.35	
**SC2 (Post 12 months)**	Cu_2_O	(211)	26.85	26.85
	Cu	(111)	29.22	28.04
		(200)	27.30	
		(220)	27.61	

**Table 4:** Comparative analysis of Cu phases and storage environments with previous reports.

S. NO.	Capping Molecule	Stability for	Oxide Planes Observed		Stored Environment	Temperature (°C)	Ref.
1	Oleylamine	1 Hour	(111), (220)		Air	230	[82]
2	AA	11 days	Not available			Not available	[83]
3	Graphene	60 days					[84]
4	PVP and AA	22 days			Water	210	[85]
5	CTAB and alkanethiols	During Drying	(110)		Vacuum	80	[58]
6	Tetraoctylammonium Bromide	Not available			n-dodecane	150	[86]
7	PVP	30 min	(110), (211), (311), (222)		Water	Not available	[87]
8	PEG	2 days	Not available		Water	Room temperature	[88]
9	PVP	1 day	(220), (110)		Air	Room temperature	[89]
10	PVP	Not available			Vacuum	200	[90]
11	PVP	20 days	Not employed		Air	180	[91]
12	Sodium metaphosphate	Not available				200	[44]
13	Not employed	30 min	(200), (111), (−111)			150	[92]
14	3-mercaptopropionic acid	7 days	Not available			Room temperature	[6]
15	AA	Not available				237	[44]
16	Lauric acid	2 day		(111), (220)		Room temperature	[21]
17	Not employed	Not available		(111)			[93]
18	SiN_x_	30 min		(111), (220)	CO	400	[94]
19	PVP	240 min		-	Methanol	182	[95]
**20**	**PVP and AA**	**12 months**		**(−111), (111), and (211)**	**Air**	**150**	**This work**

## Data Availability

All data that support the findings of this study are included in the article.

## Supplementary Materials

The following supporting information can be downloaded at: https://www.mdpi.com/article/10.3390/nano15241852/s1.

## References

[1] Narayanan K., Gnanaprakash D. (2022). Branched Gold Nanostructures Through a Facile Fructose Mediated Microwave Route. J. Clust. Sci..

[2] Kelly K.L., Coronado E., Zhao L.L., Schatz G.C. (2003). The Optical Properties of Metal Nanoparticles: The Influence of Size, Shape, and Dielectric Environment. J. Phys. Chem. B.

[3] Link S., El-Sayed M.A. (1999). Simulation of the Optical Absorption Spectra of Gold Nanorods as a Function of Their Aspect Ratio and the Effect of the Medium Dielectric Constant. J. Phys. Chem. B.

[4] Indhu A.R., Keerthana L., Dharmalingam G. (2023). Plasmonic nanotechnology for photothermal applications—An evaluation. Beilstein J. Nanotechnol..

[5] Indhu A.R., Dharanya C., Dharmalingam G. (2023). Plasmonic Copper: Ways and Means of Achieving, Directing, and Utilizing Surface Plasmons. Plasmonics.

[6] Dabera G.D.M., Walker M., Sanchez A.M., Pereira H.J., Beanland R., Hatton R.A. (2017). Retarding oxidation of copper nanoparticles without electrical isolation and the size dependence of work function. Nat. Commun..

[7] LaGrow A.P., Ward M.R., Lloyd D.C., Gai P.L., Boyes E.D. (2017). Visualizing the Cu/Cu_2_O Interface Transition in Nanoparticles with Environmental Scanning Transmission Electron Microscopy. J. Am. Chem. Soc..

[8] Chan G.H., Zhao J., Hicks E.M., Schatz G.C., Van Duyne R.P. (2007). Plasmonic Properties of Copper Nanoparticles Fabricated by Nanosphere Lithography. Nano Lett..

[9] Kim J.H., Ehrman S.H., Germer T.A. (2004). Influence of particle oxide coating on light scattering by submicron metal particles on silicon wafers. Appl. Phys. Lett..

[10] Pedersen D.B., Wang S., Liang S.H. (2008). Charge-Transfer-Driven Diffusion Processes in Cu@Cu-Oxide Core−Shell Nanoparticles: Oxidation of 3.0 ± 0.3 nm Diameter Copper Nanoparticles. J. Phys. Chem. C.

[11] Ramsey J.A., Garlick G.F.J., Roberts J.K. (1949). Theory of the oxidation of metals Some interactions of gases with metals and crystalline solids. Rep. Prog. Phys..

[12] Yang J.C., Kolasa B., Gibson J.M., Yeadon M. (1998). Self-limiting oxidation of copper. Appl. Phys. Lett..

[13] Chen C.-H., Yamaguchi T., Sugawara K.-I., Koga K. (2005). Role of Stress in the Self-Limiting Oxidation of Copper Nanoparticles. J. Phys. Chem. B.

[14] Cudennec Y., Lecerf A., Gérault Y. (1995). Synthesis of Cu(OH)_2_ and CuO by soft chemistry. Eur. J. Solid State Inorg. Chem..

[15] Cudennec Y., Lecerf A., Riou A., Gérault Y., Cudennec Y., Lecerf A., Riou A., Synthesis Y.G. (1988). Synthesis and study of sodium hydroxi-cuprate: Na_2_Cu(OH)_4_ and copper hydroxide: Cu(OH)_2_. Eur. J. Solid State Inorg. Chem..

[16] Cudennec Y., Lecerf A. (2006). The transformation of ferrihydrite into goethite or hematite, revisited. J. Solid State Chem..

[17] Azarian A., Zad A.I., Dolati A., Ghorbani M. (2007). Time dependence of the surface plasmon resonance of copper nanorods. J. Phys. Condens. Matter.

[18] He R., Wang Y.-C., Wang X., Wang Z., Liu G., Zhou W., Wen L., Li Q., Wang X., Chen X. (2014). Facile synthesis of pentacle gold–copper alloy nanocrystals and their plasmonic and catalytic properties. Nat. Commun..

[19] Plascencia G., Utigard T., Marín T. (2005). The oxidation resistance of copper-aluminum alloys at temperatures up to 1000 °C. JOM.

[20] Yamaguchi T., Kazuma E., Sakai N., Tatsuma T. (2012). Photoelectrochemical Responses from Polymer-coated Plasmonic Copper Nanoparticles on TiO_2_. Chem. Lett..

[21] Kanninen P., Johans C., Merta J., Kontturi K. (2008). Influence of ligand structure on the stability and oxidation of copper nanoparticles. J. Colloid Interface Sci..

[22] Xiong J., Wang Y., Xue Q., Wu X. (2011). Synthesis of highly stable dispersions of nanosized copper particles using l-ascorbic acid. Green Chem..

[23] Liu Y., Chu Y., Zhuo Y., Dong L., Li L., Li M. (2007). Controlled Synthesis of Various Hollow Cu Nano/MicroStructures via a Novel Reduction Route. Adv. Funct. Mater..

[24] Beltrán-Partida E., Valdez-Salas B., Valdez-Salas E., Pérez-Cortéz G., Nedev N. (2019). Synthesis, Characterization, and In Situ Antifungal and Cytotoxicity Evaluation of Ascorbic Acid-Capped Copper Nanoparticles. J. Nanomater..

[25] Biçer M., Şişman I. (2010). Controlled synthesis of copper nano/microstructures using ascorbic acid in aqueous CTAB solution. Powder Technol..

[26] Sood A., Arora V., Shah J., Kotnala R., Jain T.K. (2016). Ascorbic acid-mediated synthesis and characterisation of iron oxide/gold core–shell nanoparticles. J. Exp. Nanosci..

[27] Sreeja V., Jayaprabha K.N., Joy P.A. (2015). Water-dispersible ascorbic-acid-coated magnetite nanoparticles for contrast enhancement in MRI. Appl. Nanosci..

[28] Indhu A.R., Dharmalingam G. (2024). Microwave Prepared Oxidation Resistant Cu Microstructures with Tailored Morphologies. Chem. Afr..

[29] Zhang Y., Zhu P., Li G., Zhao T., Fu X., Sun R., Zhou F., Wong C.-P. (2014). Facile Preparation of Monodisperse, Impurity-Free, and Antioxidation Copper Nanoparticles on a Large Scale for Application in Conductive Ink. ACS Appl. Mater. Interfaces.

[30] Li Y., Yang Y., Yu A.-N., Wang K. (2016). Effects of reaction parameters on self-degradation of L-ascorbic acid and self-degradation kinetics. Food Sci. Biotechnol..

[31] Yuan J.-P., Chen F. (1998). Degradation of Ascorbic Acid in Aqueous Solution. J. Agric. Food Chem..

[32] Sadeghi B., Sadjadi M.A.S., Pourahmad A. (2008). Effects of protective agents (PVA & PVP) on the formation of silver nanoparticles. Int. J. Nanosci. Nanotechnol..

[33] del Caño R., Gisbert-González J.M., González-Rodríguez J., Sánchez-Obrero G., Madueño R., Blázquez M., Pineda T. (2020). Effective replacement of cetyltrimethylammonium bromide (CTAB) by mercaptoalkanoic acids on gold nanorod (AuNR) surfaces in aqueous solutions. Nanoscale.

[34] Aljadaani A.H.A., Al-Thabaiti S.A., Khan Z. (2021). SDS capped Cu nanorods: Photosynthesis, stability, and their catalytic activity for trypan blue oxidative degradation. J. Mater. Res. Technol..

[35] Tan M., Balela M.D. (2015). Oleylamine Assisted Synthesis of Ultralong Copper Nanowires. MATEC Web of Conferences, Proceedings of the 2015 4th International Conference on Engineering and Innovative Materials (ICEIM 2015), Penang, Malaysia, 3–4 September 2015.

[36] Jang Y., Lee N., Kim J.H., Park Y.I., Piao Y. (2018). Shape-Controlled Synthesis of Au Nanostructures Using EDTA Tetrasodium Salt and Their Photothermal Therapy Applications. Nanomaterials.

[37] Meng F., Jin S. (2012). The Solution Growth of Copper Nanowires and Nanotubes is Driven by Screw Dislocations. Nano Lett..

[38] Koczkur K.M., Mourdikoudis S., Polavarapu L., Skrabalak S.E. (2015). Polyvinylpyrrolidone (PVP) in nanoparticle synthesis. Dalton Trans..

[39] Ye J.-Y., Attard G.A., Brew A., Zhou Z.-Y., Sun S.-G., Morgan D.J., Willock D.J. (2016). Explicit Detection of the Mechanism of Platinum Nanoparticle Shape Control by Polyvinylpyrrolidone. J. Phys. Chem. C.

[40] Rónavári A., Bélteky P., Boka E., Zakupszky D., Igaz N., Szerencsés B., Pfeiffer I., Kónya Z., Kiricsi M. (2021). Polyvinyl-Pyrrolidone-Coated Silver Nanoparticles—The Colloidal, Chemical, and Biological Consequences of Steric Stabilization under Biorelevant Conditions. Int. J. Mol. Sci..

[41] Centa U.G., Mihelčič M., Sterniša M., Perše L.S. (2025). Tackling microbial adhesion to surfaces by adding mesoporous SiO_2_ nanoparticles to nanocomposite based on PVDF-HFP and PVP polymers. Surfaces Interfaces.

[42] Falqi F.H., Bin-Dahman O.A., Hussain M., Al-Harthi M.A. (2018). Preparation of Miscible PVA/PEG Blends and Effect of Graphene Concentration on Thermal, Crystallization, Morphological, and Mechanical Properties of PVA/PEG (10 wt%) Blend. Int. J. Polym. Sci..

[43] Marques A.C., Pinheiro T., Morais M., Martins C., Andrade A.F., Martins R., Sales M.G.F., Fortunato E. (2021). Bottom-up microwave-assisted seed-mediated synthesis of gold nanoparticles onto nanocellulose to boost stability and high performance for SERS applications. Appl. Surf. Sci..

[44] Wu S. (2007). Preparation of fine copper powder using ascorbic acid as reducing agent and its application in MLCC. Mater. Lett..

[45] Rehan M., Mowafi S., Aly S.A., Elshemy N.S., Haggag K. (2017). Microwave-heating for in-situ Ag NPs preparation into viscose fibers. Eur. Polym. J..

[46] Zhu Y.-J., Chen F. (2014). Microwave-Assisted Preparation of Inorganic Nanostructures in Liquid Phase. Chem. Rev..

[47] Jahan I., Erci F., Isildak I. (2021). Facile microwave-mediated green synthesis of non-toxic copper nanoparticles using Citrus sinensis aqueous fruit extract and their antibacterial potentials. J. Drug Deliv. Sci. Technol..

[48] Sreeju N., Rufus A., Philip D. (2016). Microwave-assisted rapid synthesis of copper nanoparticles with exceptional stability and their multifaceted applications. J. Mol. Liq..

[49] Tanghatari M., Sarband Z.N., Rezaee S., Larijani K. (2017). Microwave assisted green synthesis of copper nanoparticles. Bulg. Chem. Commun. Spec. Issue J..

[50] Yallappa S., Manjanna J., Sindhe M.A., Satyanarayan N.D., Pramod S.N., Nagaraja K. (2013). Microwave assisted rapid synthesis and biological evaluation of stable copper nanoparticles using T. arjuna bark extract. Spectrochim. Acta Part A Mol. Biomol. Spectrosc..

[51] Zhu H.-T., Zhang C.-Y., Yin Y.-S. (2004). Rapid synthesis of copper nanoparticles by sodium hypophosphite reduction in ethylene glycol under microwave irradiation. J. Cryst. Growth.

[52] Blosi M., Albonetti S., Dondi M., Martelli C., Baldi G. (2011). Microwave-assisted polyol synthesis of Cu nanoparticles. J. Nanoparticle Res..

[53] Galletti A.M.R., Antonetti C., Marracci M., Piccinelli F., Tellini B. (2013). Novel microwave-synthesis of Cu nanoparticles in the absence of any stabilizing agent and their antibacterial and antistatic applications. Appl. Surf. Sci..

[54] El-Berry M.F., Sadeek S.A., Abdalla A.M., Nassar M.Y. (2021). Microwave-assisted fabrication of copper nanoparticles utilizing different counter ions: An efficient photocatalyst for photocatalytic degradation of safranin dye from aqueous media. Mater. Res. Bull..

[55] Naik R., Shivashankar S.A., Bindu P.J. (2020). Microwave-assisted synthesis of copper nanoparticles: Influence of copper nanoparticles morphology on the antimicrobial activity. J. Mat. NanoSci..

[56] Zhang X., Cheng X., Yin H., Yuan J., Xu C. (2008). Preparation of needle shaped nano-copper by microwave-assisted water system and study on its application of enhanced epoxy resin coating electrical conductivity. Appl. Surf. Sci..

[57] Indhu A.R., Dharmalingam G. (2024). Oxidative and Colloidal Kinetics of Size-Controlled Copper Structures through Surface Plasmon-Regulated Examinations for Broadband Absorbance. ChemistrySelect.

[58] Chen L., Zhang D., Chen J., Zhou H., Wan H. (2006). The use of CTAB to control the size of copper nanoparticles and the concentration of alkylthiols on their surfaces. Mater. Sci. Eng. A.

[59] Aromaa J., Kekkonen M., Mousapour M., Jokilaakso A., Lundström M. (2021). The Oxidation of Copper in Air at Temperatures up to 100 °C. Corros. Mater. Degrad..

[60] Lin Y., Chen Z., Fang L., Meng M., Liu Z., Di Y., Cai W., Huang S., Gan Z. (2018). Copper nanoparticles with near-unity, omnidirectional, and broadband optical absorption for highly efficient solar steam generation. Nanotechnology.

[61] Ben-Sasson M., Lu X., Nejati S., Jaramillo H., Elimelech M. (2016). In situ surface functionalization of reverse osmosis membranes with biocidal copper nanoparticles. Desalination.

[62] Zhang X., Zhang D., Ni X., Zheng H. (2006). One-step preparation of copper nanorods with rectangular cross sections. Solid State Commun..

[63] Zhang J., Chen J., Wang Z. (2007). Surfactant-assisted preparation of single-crystalline Fe3O4 nanowires under low magnetic field. Mater. Lett..

[64] Xuan S., Wang F., Wang Y.-X.J., Yu J.C., Leung K.C.-F. (2010). Facile synthesis of size-controllable monodispersed ferrite nanospheres. J. Mater. Chem..

[65] Wiley B.J., Wang Z., Wei J., Yin Y., Cobden D.H., Xia Y. (2006). Synthesis and Electrical Characterization of Silver Nanobeams. Nano Lett..

[66] Hsieh M.-Y., Huang P.-J. (2022). Magnetic nanoprobes for rapid detection of copper ion in aqueous environment by surface-enhanced Raman spectroscopy. RSC Adv..

[67] Kang B., Tang H., Zhao Z., Song S. (2020). Hofmeister Series: Insights of Ion Specificity from Amphiphilic Assembly and Interface Property. ACS Omega.

[68] Filankembo A., Giorgio S., Lisiecki I., Pileni M.P. (2003). Is the Anion the Major Parameter in the Shape Control of Nanocrystals?. J. Phys. Chem. B.

[69] Whitehead C.B., Özkar S., Finke R.G. (2021). LaMer’s 1950 model of particle formation: A review and critical analysis of its classical nucleation and fluctuation theory basis, of competing models and mechanisms for phase-changes and particle formation, and then of its application to silver halide, semiconductor, metal, and metal-oxide nanoparticles. Mater. Adv..

[70] Park B.K., Jeong S., Kim D., Moon J., Lim S., Kim J.S. (2007). Synthesis and size control of monodisperse copper nanoparticles by polyol method. J. Colloid Interface Sci..

[71] Herring N.P., AbouZeid K., Mohamed M.B., Pinsk J., El-Shall M.S. (2011). Formation Mechanisms of Gold–Zinc Oxide Hexagonal Nanopyramids by Heterogeneous Nucleation using Microwave Synthesis. Langmuir.

[72] Yu W., Xie H., Chen L., Li Y., Zhang C. (2009). Synthesis and Characterization of Monodispersed Copper Colloids in Polar Solvents. Nanoscale Res. Lett..

[73] Borodko Y., Lee H.S., Joo S.H., Zhang Y., Somorjai G. (2010). Spectroscopic Study of the Thermal Degradation of PVP-Capped Rh and Pt Nanoparticles in H_2_ and O_2_ Environments. J. Phys. Chem. C.

[74] Averitt R.D., Westcott S.L., Halas N.J. (1999). Linear optical properties of gold nanoshells. J. Opt. Soc. Am. B.

[75] White A.H., Germer L.H. (1942). The Rate of Oxidation of Copper at Room Temperature. Trans. Electrochem. Soc..

[76] Sekhon J.S., Verma S.S. Cu, CuO, and Cu_2_O Nanoparticle Plasmons for Enhanced Scattering in Solar Cells. Proceedings of the Optical Instrumentation for Energy and Environmental Applications 2011.

[77] Harris N., Blaber M.G., Schatz G.C. (2016). Optical properties of metal nanoparticles. Encyclopedia of Nanotechnology.

[78] Jin H., Kahk J.M., Papaconstantopoulos D.A., Ferreira A., Lischner J. (2022). Plasmon-Induced Hot Carriers from Interband and Intraband Transitions in Large Noble Metal Nanoparticles. PRX Energy.

[79] Shaheen N., Nazar R., Mehmood U., Raza S.A., Iftikhar F., Naz R., Habib M.S., Rafiq M.A., Sharif S., Farooq M. (2023). Development of Polyaniline/Polyvinylpyrrolidone (PANI/PVP) Composite Films for Piezoresistive Strain-Sensing Applications. Arab. J. Sci. Eng..

[80] Oh G.-H., Hwang H.-J., Kim H.-S. (2017). Effect of copper oxide shell thickness on flash light sintering of copper nanoparticle ink. RSC Adv..

[81] Zhang H., Wen H., Peng R., He R., Li M., Feng W., Zhao Y., Liu Z. (2022). Experimental Study at the Phase Interface of a Single-Crystal Ni-Based Superalloy Using TEM. Materials.

[82] Salavati-Niasari M., Davar F. (2009). Synthesis of copper and copper(I) oxide nanoparticles by thermal decomposition of a new precursor. Mater. Lett..

[83] Asmat-Campos D., Delfin-Narciso D., Juárez-Cortijo L., Nazario-Naveda R. (2021). Influence of the volume of ascorbic acid in the synthesis of copper nanoparticles mediated by chemical pathway and its stability over time. IOP Conf. Ser. Earth Environ. Sci..

[84] Wang S., Huang X., He Y., Huang H., Wu Y., Hou L., Liu X., Yang T., Zou J., Huang B. (2012). Synthesis, growth mechanism and thermal stability of copper nanoparticles encapsulated by multi-layer graphene. Carbon.

[85] Dang T.M.D., Le T.T.T., Fribourg-Blanc E., Dang M.C. (2011). The influence of solvents and surfactants on the preparation of copper nanoparticles by a chemical reduction method. Adv. Nat. Sci. Nanosci. Nanotechnol..

[86] Ng C.H.B., Fan W.Y. (2006). Shape Evolution of Cu_2_O Nanostructures via Kinetic and Thermodynamic Controlled Growth. J. Phys. Chem. B.

[87] Dadgostar N., Ferdous S., Henneke D. (2010). Colloidal synthesis of copper nanoparticles in a two-phase liquid–liquid system. Mater. Lett..

[88] Dang C.M., Trinh C.D., Dang D.M.T., Blanc E.F. (2013). Characteristics of colloidal copper particles prepared by using polyvinyl pyrrolidone and polyethylene glycol in chemical reduction method. Int. J. Nanotechnol..

[89] Pastoriza-Santos I., Sánchez-Iglesias A., Rodríguez-González B., Liz-Marzán L.M. (2009). Aerobic Synthesis of Cu Nanoplates with Intense Plasmon Resonances. Small.

[90] Jeong S., Woo K., Kim D., Lim S., Kim J.S., Shin H., Xia Y., Moon J. (2008). Controlling the Thickness of the Surface Oxide Layer on Cu Nanoparticles for the Fabrication of Conductive Structures by Ink-Jet Printing. Adv. Funct. Mater..

[91] Lee Y., Choi J.-R., Lee K.J., Stott N.E., Kim D. (2008). Large-scale synthesis of copper nanoparticles by chemically controlled reduction for applications of inkjet-printed electronics. Nanotechnology.

[92] Choudhary S., Sarma J.V.N., Pande S., Ababou-Girard S., Turban P., Lepine B., Gangopadhyay S. (2018). Oxidation mechanism of thin Cu films: A gateway towards the formation of single oxide phase. AIP Adv..

[93] Curtis A.C., Duff D.G., Edwards P.P., Jefferson D.A., Johnson B.F.G., Kirkland A.I., Wallace A.S. (1988). Preparation and structural characterization of an unprotected copper sol. J. Phys. Chem..

[94] Nilsson S., El Berch J.N., Albinsson D., Fritzsche J., Mpourmpakis G., Langhammer C. (2023). The Role of Grain Boundary Sites for the Oxidation of Copper Catalysts during the CO Oxidation Reaction. ACS Nano.

[95] Cuya Huaman J.L., Urushizaki I., Jeyadevan B. (2018). Large-Scale Cu Nanowire Synthesis by PVP-Ethylene Glycol Route. J. Nanomater..

