# Supplementary material for: A Focus on Thermal Durability and Oxidation Resistance and Morphology of Polymer Capped Copper Particles Through a Synthesis-Driven, Precursor-Influenced Approach
Source: Nanomaterials (Basel). 2025 Dec 10;15(24):1852. doi: 10.3390/nano15241852 (PMC12735937; doi:10.3390/nano15241852)
Supplement: Supplementary file 1 [file nanomaterials-15-01852-s001.zip › nanomaterials-3996371-supplementary.pdf]

A Focus on Thermal Durability and Oxidation Resistance and Morphology of polymer capped copper particles through a synthesis-driven, precursor-influenced approach

A.R. Indhu[1], Manickam Minakshi, R. Sivasubrmanaian[3], GnanaprakashDharmalingam[4,*]

[1] Plasmonic Nanomaterials Laboratory, Department of Nanoscience and Technology, PSG Institute of Advanced Studies, Coimbatore, Tamil Nadu, India

[2] College of Science, Health, Engineering and Education, Murdoch University, Perth, WA, Australia

[3] Department of Chemistry, Amrita School of Physical Sciences, Amrita Vishwa Vidyapeetham, Amaravati, Andhra Pradesh, India

[4] Department of Physics and Nanotechnology, SRM Institute of Science and Technology, Kattankulathur, , Chennai, Tamil Nadu, India

* corresponding author email: gnanaprd@srmist.edu.in. Ph: +91 8300188946

Supplementary Information

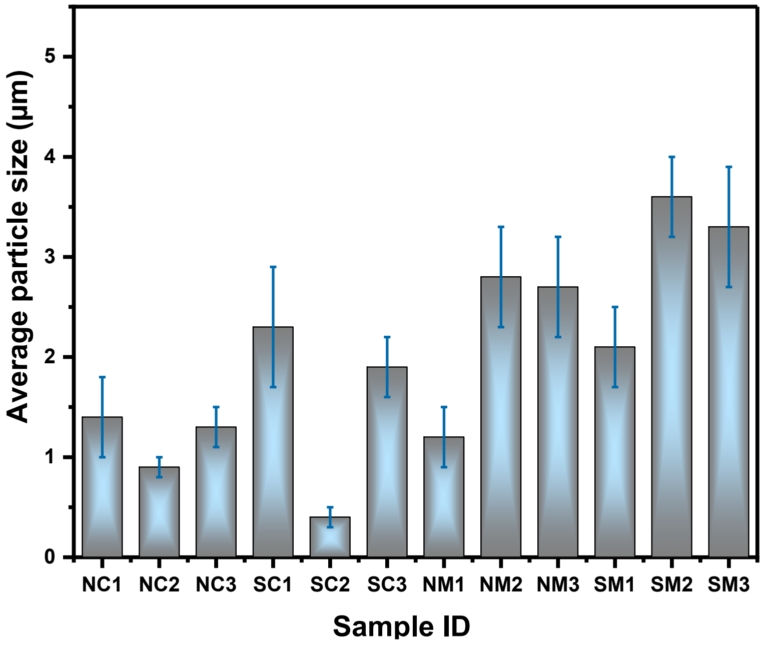

[OFFICIAL]

Figure S1 Average particle sizes with standard deviations of copper nanoparticles synthesized via chemical reduction and microwave-assisted methods using nitrate and sulphate precursors. Comparative analysis highlights the influence of precursor chemistry and synthesis technique on particle size distribution.

Figure S2 Relative intensities derived from X-ray diffraction (XRD) patterns of copper

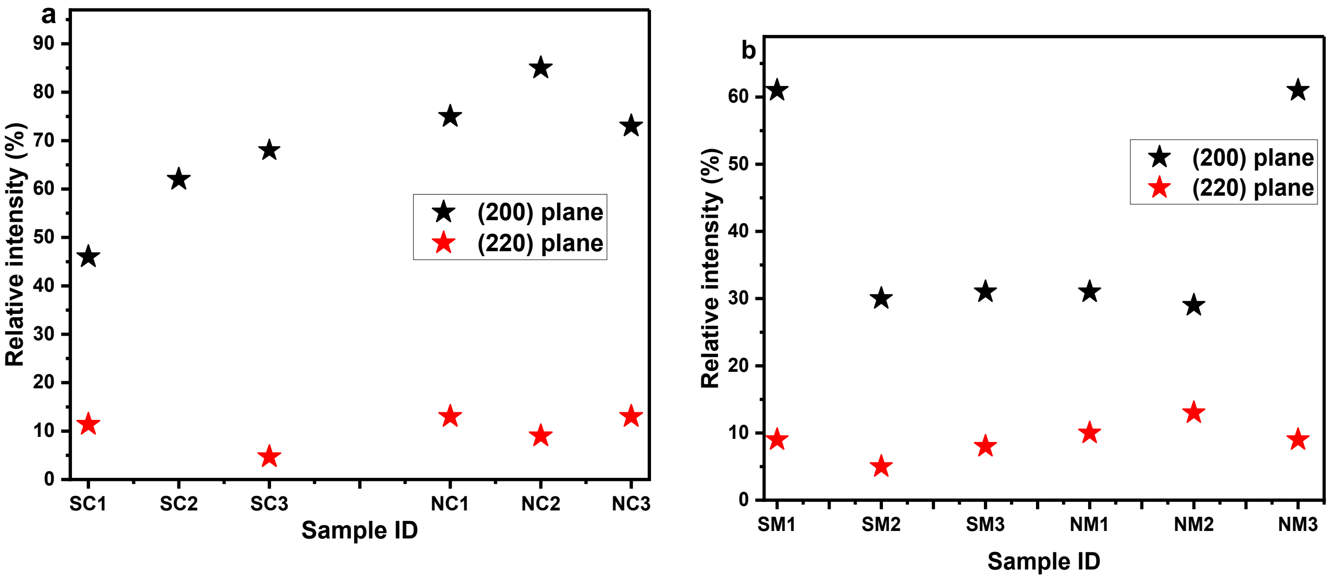

nanoparticles synthesized from nitrate and sulphate precursors immediately after synthesis. (a) Chemically reduced samples; (b) Microwave-assisted samples. The data illustrate the influence of precursor type and synthesis method on crystallographic features and phase composition.

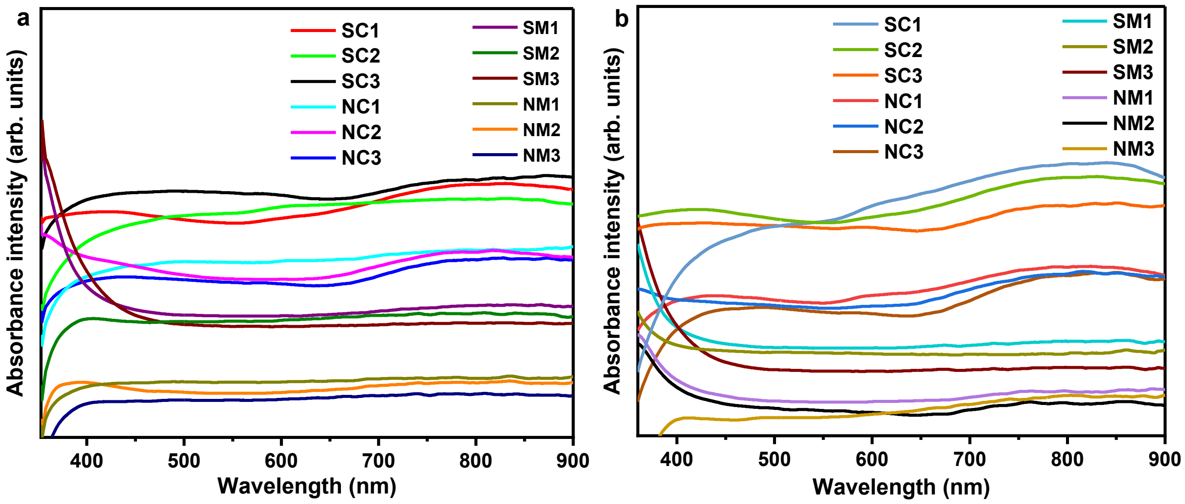

Figure S3 UV–Vis absorption spectra of copper nanoparticle samples synthesized using nitrate and sulphate precursors at varying concentrations of polyvinylpyrrolidone (PVP). (a) Supernatant fractions; (b) Precipitated fractions. The spectra reveal the effect of precursor type and PVP concentration on nanoparticle dispersion and optical properties.

[OFFICIAL]

Table S1. Comparative crystallite sizes of copper nanoparticles synthesized via microwave-assisted (MW) and chemical reduction (CR) methods, evaluated before and after thermal exposure. The data highlight the impact of synthesis technique and thermal treatment on crystallite stability and growth.

**Samples**		*CR*						*MW*					
		NC1	*SC1*	*NC2*	*SC2*	*NC3*	*SC3*	*NM1*	*SM1*	*NM2*	*SM2*	*NM3*	*SM3*
**Crystallite size (nm)**	**BT**	39.89	39.38	**Cu** 31.32<br>**CuO** 16.21 (-111)<br>18.18 (111)	**Cu** 28.48<br>**Cu_2O** 22.02 (211)	37.38	36.56	38.15	36.56	39.89	36.56	38.15	36.56
	**AT**	38.15	35.10	**Cu** 30.22<br>**CuO** 13.81 (-111)<br>15.15 (111)	**Cu** 26.56<br>**Cu_2O** -	36.56	31.34	38.15	38.15	33.75	36.56	38.15	38.15
**% of Crystallinity changes**		-4	-10	**Cu**-3.5<br>**CuO** (-111)-14<br>(111)-16	Cu-6.7	-2	-14	0	4	-15	0	0	-4

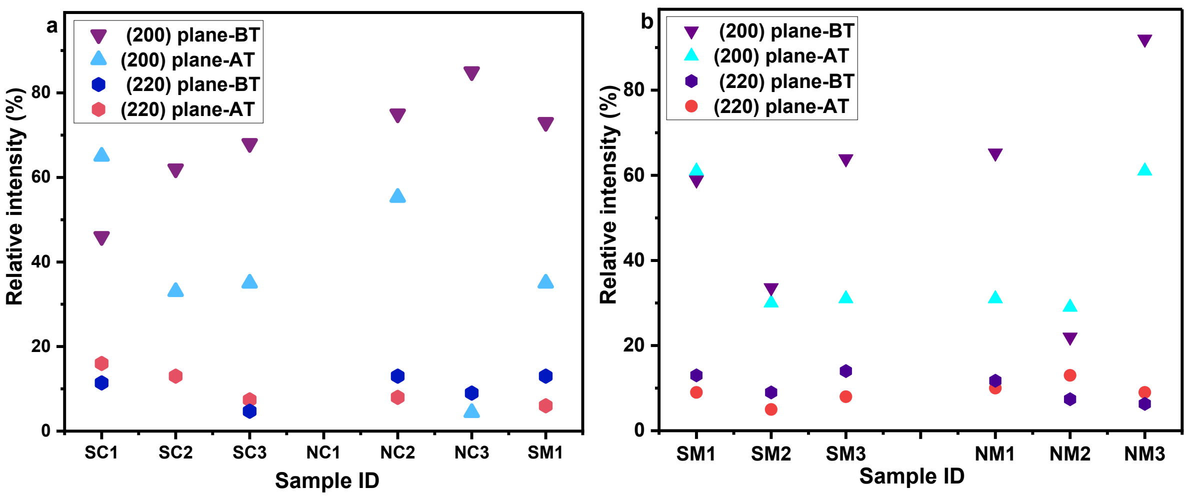

Figure S4 Relative diffraction intensities of copper nanoparticle samples before and after thermal exposure, normalized to the (111) crystallographic plane. (a) Chemically reduced (CR) samples; (b) Microwave-assisted (MW) samples.

[OFFICIAL]

Table S2. Comparative FTIR peak positions of copper nanoparticle samples synthesized via chemical reduction and microwave-assisted methods, evaluated before and after thermal exposure.

**Functional groups**	**Sample ID**			
	**NC1-BT**	**NC1-AT**	**SC1-BT**	**SC1-AT**
C=O	1735	1738	1740	1747
C-H	2922	2933	2929	2929
C-N	-	1086	-	1096
CH_2	1456	-	1519	1524
**Functional groups**	**Sample ID**			
	**NC2-BT**	**NC2-AT**	**SC2-BT**	**SC2-AT**
C=O	1734	-	1735	-
C-H	2921	2921	2930	2943
C-N	-	1082	1021	-
CH_2	1448	1516	1459	1459
**Functional groups**	**Sample ID**			
	**NC3-BT**	**NC3-AT**	**SC3-BT**	**SC3-AT**
C=O	1735	-	1730	1750
C-H	2918	2934	2924	2931
C-N	1022	1026	1017	1026
CH_2	1451	1536	1455	1513
**Functional groups**	**Sample ID**			
	**NM1-BT**	**NM1-AT**	**SM1-BT**	**SM1-AT**
C=O	1648	1746	1730	1747
C-H	2925	2930	2924	2928
C-N	-	-	1017	-
CH_2	1547	1519	1455	1519
**Functional groups**	**Sample ID**			
	**NM2-BT**	**NM2-AT**	**SM2-BT**	**SM2-AT**
C=O	1751	1751	1731	1748
C-H	2910	2923	2925	2953
C-N	-	-	1023	1023
CH_2	1550	1525	1510	1519
**Functional groups**	**Sample ID**			
	**NM3-BT**	**NM3-AT**	**SM3-BT**	**SM3-AT**
C=O	1745	-	1731	1748
C-H	2906	2934	2925	2953
C-N	1018	1026	1023	1023
CH_2	1455	1536	1510	1519

[OFFICIAL]

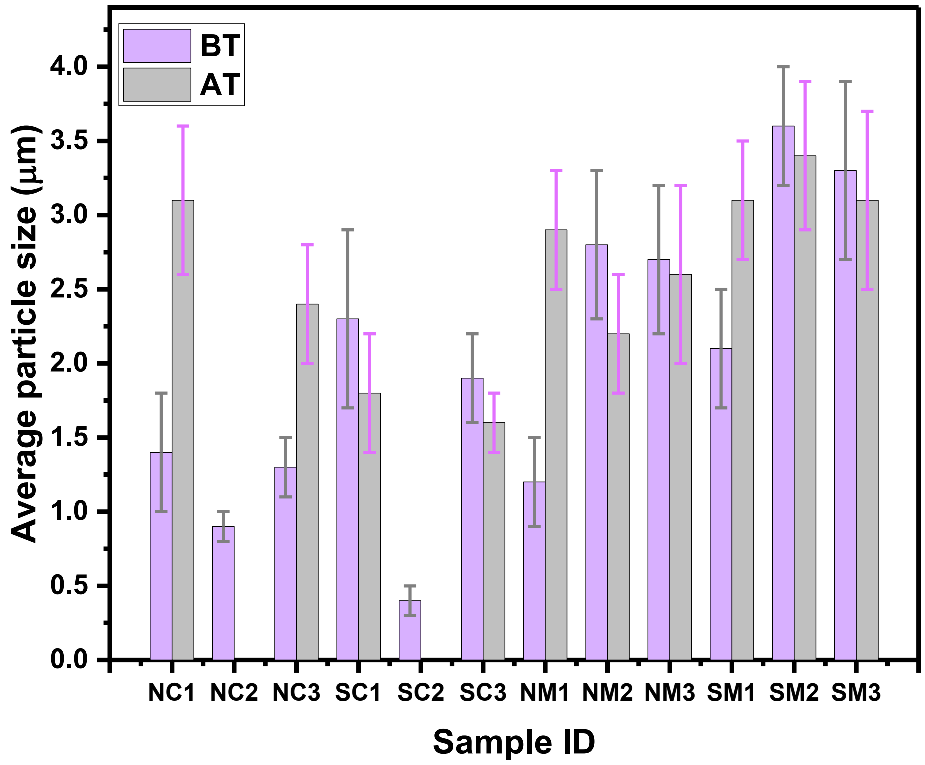

Figure S5 Comparative average particle sizes of copper nanoparticles, calculated from SEM images, with corresponding standard deviations (SD), before and after thermal exposure.

[OFFICIAL]
